# Targeting immunoliposomes to EGFR-positive glioblastoma

**DOI:** 10.1016/j.esmoop.2021.100365

**Published:** 2022-01-05

**Authors:** B. Kasenda, D. König, M. Manni, R. Ritschard, U. Duthaler, E. Bartoszek, A. Bärenwaldt, S. Deuster, G. Hutter, D. Cordier, L. Mariani, J. Hench, S. Frank, S. Krähenbühl, A. Zippelius, C. Rochlitz, C. Mamot, A. Wicki, H. Läubli

**Affiliations:** 1 Division of Oncology, Department of Internal Medicine, University Hospital Basel, Basel, Switzerland; 2 University of Basel, Basel, Switzerland; 3 Department of Biomedicine, University Hospital and University of Basel, Basel, Switzerland; 4 Division of Clinical Pharmacology, University Hospital Basel, Basel, Switzerland; 5 Hospital Pharmacy, University Hospital Basel, Basel, Switzerland; 6 Neurosurgery, University Hospital Basel, Basel, Switzerland; 7 Institute of Pathology, University Hospital Basel, Basel, Switzerland; 8 Division of Medical Oncology, Cantonal Hospital, Aarau, Switzerland

**Keywords:** nanomedicine, blood–brain barrier, targeted therapy, pharmacokinetic, cerebrospinal fluid

## Abstract

**Background:**

We assessed the capacity of epidermal growth factor receptor (EGFR)-targeted immunoliposomes to deliver cargo to brain tumor tissue in patients with relapsed glioblastoma harboring an EGFR amplification. We aimed to assess the tolerability and effectiveness of anti-EGFR immunoliposomes loaded with doxorubicin (anti-EGFR ILs-dox) in glioblastoma multiforme patients.

**Patients and methods:**

Patients with EGFR-amplified, relapsed glioblastoma were included in this phase I pharmacokinetic trial. Patients received up to four cycles of anti-EGFR ILs-dox. Twenty-four hours later, plasma and cerebrospinal fluid (CSF) samples were obtained. In addition, we also treated three patients with anti-EGFR ILs-dox before resection of their relapsed glioblastoma. Doxorubicin concentrations were measured in plasma, CSF, and tumor tissue. Safety and efficacy parameters were also obtained.

**Results:**

There were no or negligible levels of doxorubicin found in the CSF demonstrating that anti-EGFR ILs-dox are not able to cross the blood–brain barrier (BBB). However, significant levels were detected in glioblastoma tissue 24 h after the application, indicating that the disruption of BBB integrity present in high-grade gliomas might enable liposome delivery into tumor tissue. No new safety issues were observed. The median progression-free survival was 1.5 months and the median overall survival was 8 months. One patient undergoing surgery had a very long remission suggesting that neoadjuvant administration may have a positive effect on outcome.

**Conclusions:**

We clearly demonstrate that anti-EGFR-immunoliposomes can be targeted to EGFR-amplified glioblastoma and cargo—in this case doxorubicin—can be delivered, although these immunoliposomes do not cross the intact BBB. (The GBM-LIPO trial was registered as NCT03603379).

## Highlights

• Human pharmacokinetic and pharmacodynamic data for EGFR-targeted immunoliposomes.
• Demonstration of delivery of immunoliposomes to glioblastoma tissue.
• EGFR as a target to deliver drug-containing nanoparticles to glioma tissue.

## Introduction

Glioblastoma is a malignant brain tumor with a poor prognosis for almost all patients.^1^, ^2^, ^3^, ^4^ The current standard treatment option for glioblastoma patients is tumor resection followed by adjuvant radiochemotherapy with 60 Gy and the alkylating agent temozolomide.^3^ Recently, the addition of tumor-treating fields to radiochemotherapy led to an improvement of survival prognosis.^5^^,^^6^ However, most glioblastoma patients will experience relapse after 12-15 months.^1^, ^2^, ^3^ The treatment of relapsed glioblastoma is challenging because of only few treatment options.^7^^,^^8^ Anti-angiogenic therapy with bevacizumab can lead to disease stabilization and regression of glioblastoma in some patients, but there is no evidence for prolonged survival.^4^^,^^7^^,^^9^ Lomustin is also used in patients with relapsed glioblastoma, but in most trials median survival is poor with only 4-6 months.^4^^,^^7^^,^^10^

The amplification and mutation of the epidermal growth factor receptor (EGFR) is among the most frequently found genetic aberrations in glioblastoma, which can be detected in about 40%-50% of glioblastomas.^11^^,^^12^ Amplification of EGFR is often accompanied by the appearance of a variant form of EGFR called variant III (EGFRvIII).^12^ Targeting EGFR in glioblastoma patients with tyrosine kinase inhibitors used for the treatment of EGFR-mutated lung cancer has not shown convincing efficacy, likely because of the lack of sensitizing mutations and intratumoral heterogeneity of EGFR expression.^13^^,^^14^ However, the high frequency of EGFR amplification remains an interesting target. EGFR-directed therapy in glioblastoma has included treatment with the antibody-drug conjugate depatuxizumab mafodotin (Depatux-M) composed of the EGFR immunoglobulin G1 monoclonal antibody depatuxizumab coupled to the tubulin inhibitor monomethyl auristatin F (MMAF).^11^ In the INTELLANCE-2/EORTC 1410 trial, combination of depatuxizumab–MMAF has shown some efficacy in combination with temozolomide,^11^ but the first-line INTELLANCE-1 trial was prematurely stopped due to a lack of survival benefit. EGFR and in particular EGFRvIII have been also used to redirect immune cells to glioblastoma.^15^^,^^16^

A possible approach to reach high concentrations of antitumor agents in the tumor tissue is the use of nanocarriers/nanoparticles that are ‘filled’ with cytotoxic drugs linked to a targeting agent such as antibodies or antibody fragments.^17^^,^^18^ While a range of different nanocarriers have been tested in preclinical models (and a few of them clinically),^19^ lipid carriers remain the most advanced and clinically relevant nanoparticles in oncology. There are several formulations of lipid-based nanocarriers,^17^^,^^18^ and the most frequently analyzed carriers are liposomes (closed phospholipid bilayers) and micelles (normal phase, oil-in-water micelles).^17^^,^^18^ Lipid nanocarriers have an extensive carrying capacity, which is three to four orders of magnitude higher than drug conjugates, which typically comprise 1-6 drug molecules per monoclonal antibody.^19^^,^^20^ To target EGFR, immunoliposomes were developed and linked to the antigen-binding fragment (Fab) of the cetuximab antibody.^20^ The Fab was covalently conjugated to maleimide groups at the termini of DSPE-PEG (1, 2-Distearoyl-sn-glycero-3-phosphoethanolamine-Poly ethylene glycol) chains, which reside in the lipid bilayer.^20^ These anti-EGFR doxorubicin-loaded immunoliposomes (anti-EGFR ILs-dox) displayed highly efficient binding and internalization in a panel of EGFR or EGFRvIII overexpressing cancer cell lines, as indicated by fluorescence microscopy and fluorescence-activated cell sorter.^17^^,^^18^ Anti-EGFR ILs-dox were tested in a phase I trial in solid tumors.^21^ Twenty-nine patients were treated with anti-EGFR ILs-dox, with one patient experiencing a complete remission and one patient with partial response.^21^ Patients with glioblastoma were excluded from this study and the distribution of nanoparticles in the central nervous system (CNS) has not yet been studied.

In the GBM-LIPO trial, we treated patients with relapsed glioblastoma harboring an EGFR amplification with anti-EGFR ILs-dox and assessed its pharmacokinetics within the CNS compartment.

## Patients and methods

### Patients

We included patients with histologically proven, EGFR-amplified glioblastoma after first or later relapse. EGFR amplification was identified by comparative genomic hybridization (CGH). The definition of EGFR amplification was the ratio of EGFR/centromere chromosome 7 above 0.15. Patients were required to have an Eastern Cooperative Oncology Group performance status of 0-2 (on a five-point scale, with higher numbers indicating greater disability) and measurable disease according to Response Assessment in Neuro-Oncology (RANO) criteria;^22^ a recently obtained or archival tumor specimen; and adequate hematologic, hepatic, and renal function. Key exclusion criteria included the following: cardiopulmonary dysfunction (including heart failure of New York Heart Association class II or higher or a history of a reduction in the left ventricular ejection fraction to <40% with previous therapy) and life expectancy of <2 months.

### Trial design

This was a pharmacokinetic phase I trial (GBM-LIPO trial) to assess the target concentration of doxorubicin delivered to the glioblastoma tissue and compare it to cerebrospinal fluid (CSF) and plasma levels (see trial protocol in the Supplementary Material, available at https://doi.org/10.1016/j.esmoop.2021.100365). Anti-EGFR ILs-dox were administered intravenously at a dose of 50 mg/m^2^ for a maximum of four cycles. Each treatment cycle was 28 days. The dose of 50 mg/m^2^ was based on a previous phase I clinical study of anti-EGFR ILs-dox in patients with solid tumors.^21^ All patients included in the study were discussed at an interdisciplinary neuro-oncology tumor board and patients who were planned for resection of relapsed disease received anti-EGFR ILs-dox 24 h before surgery. The trial was conducted at two sites in Switzerland (University Hospital Basel and Cantonal Hospital Aarau).

### Trial oversight

The trial was conducted in accordance with the ethics principles of the Declaration of Helsinki and with the Good Clinical Practice guidelines defined by the International Council for Harmonisation. The trial protocol was approved by the responsible independent ethics committee (EKNZ) and the Swiss Agency for Therapeutic Products (Swissmedic). All patients provided written informed consent. The trial was designed by the principal investigators and registered with ClinicalTrials.gov (NCT03603379).

### Endpoints and assessments

The main endpoints included concentration of anti-EGFR ILs-dox in plasma, the CSF, and glioblastoma tissue (if the patient had resection of the relapse). Further endpoints included adverse events graded according to the Common Terminology Criteria for Adverse Events, version 5.0, tumor response measured by magnetic resonance imaging of the brain and assessed by investigators according to RANO criteria for high-grade glioma, progression-free survival (PFS) (time from registration to tumor progression, relapse or death whichever occurred first), and overall survival (time from registration to death of any cause). Response was assessed as per protocol after 8 weeks and 16 weeks and thereafter within clinical routine.

### Production EGFR-targeting immunoliposomes

Anti-EGFR ILs-dox were produced as previously described.^21^ Briefly, immunoliposomes were prepared with commercially available pegylated liposomal doxorubicin (Caelyx, Janssen Pharmaceuticals, Beerse, Belgium) and the antigen-binding fragment of the EGFR-binding antibody clone C225 [Cetuximab (Erbitux), Merck, Darmstadt, Germany]. The antigen-binding fragment (Fab′) of cetuximab was covalently conjugated to the maleimide groups at the termini of pegylated distearoylphosphatidylethanolamine chains. Separation, purification, and concentration steps during antibody modification were carried out by fast protein liquid chromatography size exclusion and tangential flow filtration.

### Methylation-based classification of glioblastoma subtype

Whole-genome methylation analysis was carried out on the Illumina Infinium HumanMethylation450 BeadChip (450k) arrays according to the manufacturer’s instructions (Illumina, San Diego, CA). Tumor type and chromosomal copy number changes were interrogated as published by Capper and colleagues.^23^ In addition, EGFR amplification was determined by CGH.

### LC-MS/MS analysis of doxorubicin in plasma, tissue, and CSF samples

Doxorubicin was analyzed by liquid chromatography tandem mass spectrometry (LC-MS/MS). Doxorubicin and the internal standard, daunorubicin, were detected by multiple reaction monitoring in the positive ionization mode. Doxorubicin and daunorubicin were separated on a pentafluorophenyl analytical column [Luna 3 μm PFP(2) 50 × 2.0 mm, Phenomenex, Torrance, CA]. Plasma and CSF samples of 50 μl were extracted with 150 μl acetonitrile containing 100 ng/ml daunorubicin. Samples were vortex mixed for 30 s and centrifuged for 30 min at 3220*g* and 10°C (5810R, Eppendorf, Hamburg, Germany). Glioblastoma tissue (20 mg/ml) was homogenized and extracted with a mixture of acetonitrile : water (8 : 2 v/v) (Precellys Evolution, Betrin Technologies, Rockville, MD). An aliquot of 2 μl plasma supernatant or 20 μl of liquor supernatant and glioblastoma extract was injected into the LC-MS/MS system. Doxorubicin calibration lines were prepared in blank human plasma including a concentration range from 10 to 10 000 ng/ml. Calibrations were prepared in Ringer solution (Bichsel, Interlaken, Switzerland) supplemented with 3% bovine serum albumin (BSA) (Sigma-Aldrich, Buchs, Switzerland) for liquor measurements (0.25-50 ng/ml) and in blank glioblastoma tissue extracts (0.25-250 ng/ml) to estimate the doxorubicin concentration in glioblastoma. Unknown doxorubicin concentrations were calculated by linear regression of the doxorubicin concentration (x) and the peak area ratio of doxorubicin to daunorubicin (y). The regression line was weighted by 1/x^2^. Samples above the upper limit of quantification were diluted with blank matrix. Quality control samples at low, medium, and high doxorubicin concentration were included in each analytical run to review the accuracy and precision of the method. Analyst 1.6.2 (Sciex, Concord, Canada) was used to operate the LC-MS/MS system and to quantitate the doxorubicin concentration in unknown samples.

### Tissue preparation and staining for image mass cytometry (IMC)

Tissue samples were formalin-fixed and paraffin-embedded at the University Hospital of Basel. Sections were baked for 1 h at 60°C, dewaxed in fresh xylene for 20 min, and rehydrated in a graded series of alcohol (100%, 95%, 80%, 70%; 5 min each). Antigen retrieval was carried out in IHC Antigen Retrieval Solution pH 9 (Invitrogen) for 30 min in a 95°C water bath. Sections were cooled and then immediately blocked with 3% BSA for 45 min at room temperature. Samples were stained for 5 h at room temperature using the Maxpar Human Immune Activation IMC Panel Kit (Fluidigm, South San Francisco, CA) in combination with the Maxpar Human Tumor-Infiltrating Lymphocytes IMC Panel Kit (Fluidigm) and metal-conjugated anti-Vimentin (^143^Nd, clone D21H3, Fluidigm). Tissue sections were washed twice with 0.05% Tween-20 in Maxpar PBS (Fluidigm) before staining with Intercalator-Ir (Fluidigm) for 30 min at room temperature. Slides were then washed with Maxpar water for 5 min and let air-dry for at least 20 min at room temperature before acquisition.

### IMC

Analysis of the tumor microenvironment after surgery was carried out using IMC. Images were acquired using a Hyperion Imaging System coupled to a Helios Mass Cytometer (Fluidigm). Five to seven 1 × 1 mm regions of interest per section were defined and laser-ablated in a rastered pattern at 200 Hz. Images were visualized using the MCD Viewer Software (Fluidigm). Final figures were generated using OMERO (htts://www.openmicroscopy.org/omero/figure/). Cell segmentation and cell classification were carried out using QuPath software (https://qupath.github.io/).

### Statistics

We list individual patient characteristics and safety without summarizing statistics, because of the small sample size. Anti-EGFR ILs-dox concentration levels in plasma, CSF, and tumor tissue are summarized using descriptive statistics.

## Results

### Trial population

**Figure 1:**
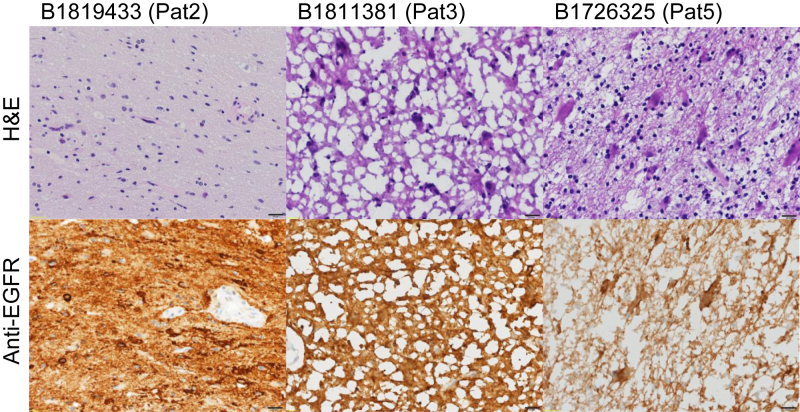
**Recurrent tumor biopsies.** Top row: Hematoxylin–eosin (H&E)-stained formalin-fixed tissue; note that B1811381 has cryoartifacts. B1726325 has low levels of residual tumor cells in this biopsy. Bottom row: Anti-epidermal growth factor receptor (EGFR) staining. Black bars represent 20 μm.

We enrolled nine eligible patients with relapsed glioblastoma and EGFR amplification. The median age of the patients at glioblastoma diagnosis was 59 years (range, 44-64 years); seven patients were male. All patients were diagnosed with glioblastoma without isocitrate dehydrogenase mutation and no loss of heterozygosity of 1p/19q (Table 1). Most glioblastomas could be stratified into receptor tyrosine kinase II and mesenchymal subtypes by methylation profiling; however, unequivocal methylation class assignment was not possible in one case. EGFR amplification was determined by CGH and all patients had at least an amplification of two or more compared to the centromere of chromosome 7. All but two patients had one previous systemic chemotherapy with temozolomide. Patients relapsed at a median of 15.3 months after primary tumor resection. One patient underwent two re-resections followed by temozolomide maintenance after the first and bevacizumab maintenance after the second relapse. One patient had received two previous treatment lines (Table 2). In three patients (patient 2, 3, and 5), anti-EGFR ILs-dox were administered 24 h before resection of tumor relapse, and in those cases doxorubicin levels were assessed in tumor tissue, in addition to plasma and CSF samples. Analysis of tumor tissue of the three patients who underwent surgery confirmed malignancy and the presence of EGFR amplification. In two patients, only few remaining cells were present (Figure 1). EGFR was still expressed (Figure 1). The amplification of EGFR was still present as determined by CGH. In six patients, surgical resection was not deemed meaningful, and plasma and CSF samples were collected 24 h after application of anti-EGFR ILs-dox to analyze the general concentration in the two compartments (plasma and CSF).

**Table 1:** Patient and disease characteristics

Patient	Age at diagnosis, years	Gender	EGFR amplification	IDH mutation	LOH 1p/19q	Methylation class	MGMT-promoter methylation
1	59	Male	Yes	IDH wt	No	RTK II	Methylated
2	44	Female	Yes	IDH wt	No	RTK II	Not methylated
3	50	Male	Yes	IDH wt	No	RTK II	Not methylated
4	64	Male	Yes	IDH wt	No	RTK II	Not methylated
5	60	Female	Yes	IDH wt	No	RTK II	Methylated
6	63	Male	Yes	IDH wt	No	Mesenchymal	Methylated
7	61	Male	Yes	IDH wt	No	Unclear	Not methylated
8	58	Male	Yes	IDH wt	No	Mesenchymal	Not methylated
9	50	Male	Yes	IDH wt	No	Mesenchymal	Methylated

EGFR, epidermal growth factor receptor; IDH, isocitrate dehydrogenase; LOH 1p/19q, loss of heterozygosity on chromosome 1 (1p) and 19 (19q); MGMT, O6-methylguanine-DNA methyltransferase; RTK, receptor tyrosine kinase; wt, wild type.

**Table 2:** Treatment lines and response

Patient	Primary resection	RCT with TMZ (dose and fraction of radiotherapy), TMZ maintenance (number of TMZ cycles), TTF	PFS1	Re-resection at first relapse	Second-line treatment (PFS)	Third-line treatment (PFS)	Fourth-line treatment (PFS)	Fifth-line treatment (PFS)	OS
1	Yes	Yes (60 Gy, 2 Gy) yes (6) yes	13.5	Yes	**Anti-EGFR** **ILs-dox** **(1.6)**	Beva (2.3)	No	No	21.0
2	Yes	Yes (60 Gy, 2 Gy) yes (2) yes	5.9	Yes^a^	**Anti-EGFR** **ILs-dox (4.1)**	Nivo + Beva (2.0)	Nivo + Ribo (0.6)	No	16.0
3	Yes	Yes (60 Gy, 2 Gy) yes (6) no	9.3	Yes^a^	**Anti-EGFR** **ILs-dox (1.68)**	Rego (0.9)	Depatux-M + TTF (3.5)	Beva + TTF (2.0)	17.6
4	Yes	Yes (39.9 Gy, 2.66 Gy) yes (6) no	9.4	No	**Anti-EGFR** **ILs-dox (0.8)**	Beva (1.9)	No	No	13.5
5	Yes	Yes (60 Gy, 2 Gy) yes (6) no	20.0	Yes^a^	**Anti-EGFR** **ILs-dox (17.9)**	No	No	No	41.9
6	Yes	Yes (60 Gy, 2 Gy) yes (6) no	14.1	Yes	Tem (2.6)	Re-Resection + Beva (5.9)	**Anti-EGFR** **ILs-dox (13.9)**	Beva + RT (3.9)	40.0
7	Yes	Yes (60 Gy, 2 Gy) yes (6) yes	11.8	Yes	Lom (3.4)	Beva (2.0)	**Anti-EGFR** **ILs-dox (1.9)**	No	31.2
8	Yes	Yes (60 Gy, 2 Gy) yes (6) no	11.4	No	**Anti-EGFR** **ILs-dox (1.1)**	Beva (2.2)	No	No	18.8
9	Yes	Yes (39.9 Gy, 2.66 Gy) yes (6) no	20.0	No	**Anti-EGFR** **ILs-dox (1.5)**	Tem (2.8)	Lom (2.0)	Beva (1.2)	30.4

Anti-EGFR ILs-dox, anti-EGFR immunoliposomes loaded with doxorubicin; Beva, bevacizumab; Depatux-M, depatuxizumab mafodotin (ABT-414); Lom, lomustine; Nivo, nivolumab; OS, overall survival (in months), calculated from primary resection until death; PFS, progression-free survival (in months), calculated from treatment start at each respective treatment line until evidence of recurrence on magnetic resonance imaging; PFS1, progression-free survival (in months), calculated from primary resection until first recurrence; RCT, radiochemotherapy with temozolomide; Rego, regorafenib; Ribo, ribociclib; RT, radiotherapy; TMZ, temozolomide; TTF, tumor-treating fields.

a Re-resection after administration of anti-EGFR ILs-dox (anti-EGFR immunoliposomes loaded with doxorubicin).

### Pharmacokinetics

The mean plasma concentration of doxorubicin assessed 24 h after administration of anti-EGFR ILs-dox was 15 805 ng/ml (range, 10 394-24 021 ng/ml) (Table 3). The concentration of doxorubicin in CSF was <1 ng/ml in all patients, with no detectable level of doxorubicin (<0.1 ng/ml) in three out of nine patients. For two patients who underwent surgery, the CSF doxorubicin concentration was not assessable because of contamination with blood. For the three patients who underwent surgery of relapse, the doxorubicin level in tumor tissue was 180 ng/g, 310 ng/g, and 3730 ng/g tumor tissue, respectively. From two of these patients, tissue from the periphery of the tumor was available. Doxorubicin levels were lower in these cases (central: 3730 ng/g versus periphery: 360 ng/g; and central: 310 ng/g versus periphery: 110 ng/g).

**Table 3:** Doxorubicin concentration in the blood, cerebrospinal fluid (CSF), and tumor tissue

Patient	Doxorubicin concentration in blood (ng/ml)	Doxorubicin concentration in CSF (ng/ml)	Doxorubicin concentration in glioblastoma tissue (ng/g tumor)
1	17 156	0.14	Not applicable
2	10 394	No suitable material^a^	3730
3	13 799	0.94	310
4	11 907	<0.1	Not applicable
5	13 268	No suitable material^a^	180
6	17 498	0.16	Not applicable
7	20 796	0.13	Not applicable
8	13 405	<0.1	Not applicable
9	24 021	<0.1	Not applicable

a Cerebrospinal fluid (CSF) in patients undergoing surgery was contaminated by blood and was not usable for the analysis.

### Safety

All adverse events that occurred during treatment (up to 30 days after treatment) are summarized in Table 4. No grade 4 or 5 adverse events occurred. One patient experienced a grade 3 pneumonitis. In this patient, anti-EGFR ILs-dox were discontinued after one application and high-dose corticosteroids immediately initiated. Pulmonary function recovered completely after treatment with corticosteroids. There were two cases of febrile neutropenia requiring hospital admission and intravenous antibiotics treatment. Both patients recovered from febrile neutropenia without sequelae. No post-operative infections occurred in the three patients who underwent surgical resection.

**Table 4:** Adverse events of all patients

Patient	Adverse events	Grade	Related to study drug	Measures taken
1	Pneumonitis	3	Yes	IV steroids, study termination
1	Infusion reaction	2	Yes	IV steroids, antihistamines
2	Infusion reaction	1	Yes	Antihistamines
2	Respiratory infection	2	No	Antipyretics
3	None	—	—	—
4	None	—	—	—
5	Febrile neutropenia	3	Yes	IV antibiotics, hospital admission
5	Fever	1	Yes	Oral antibiotics
6	None	—	—	—
7	Febrile neutropenia	3	Yes	IV antibiotics, hospital admission
8	None	—	—	—
9	None	—	—	—

IV, intravenous.

### Clinical outcome

Efficacy of anti-EGFR ILs-dox was assessed in all treated patients (Table 2). Of six patients not receiving surgery, none had a documented intracranial response according to RANO criteria. The median PFS was 1.5 months [95% confidence interval (CI) 1.3-not applicable (NA)]. Most patients (*n* = 5) progressed after two cycles. Two patients who underwent surgery experienced progression after 3.7 and 16.4 months, respectively, which resulted in an overall survival of 8.4 and 19.2 months. The median overall survival for all patients was 8 months (95% CI 6.3 months-NA). Most patients had subsequent antitumor therapies including treatment with bevacizumab (*n* = 8), and one patient received regorafenib and the EGFR-targeting agent Depatux-M. However, the patient with longest survival had no subsequent therapy and the progression occurred only after 16.4 months.

### Immune cell composition of glioblastoma in patients treated with anti-EGFR ILs-dox

**Figure 2:**
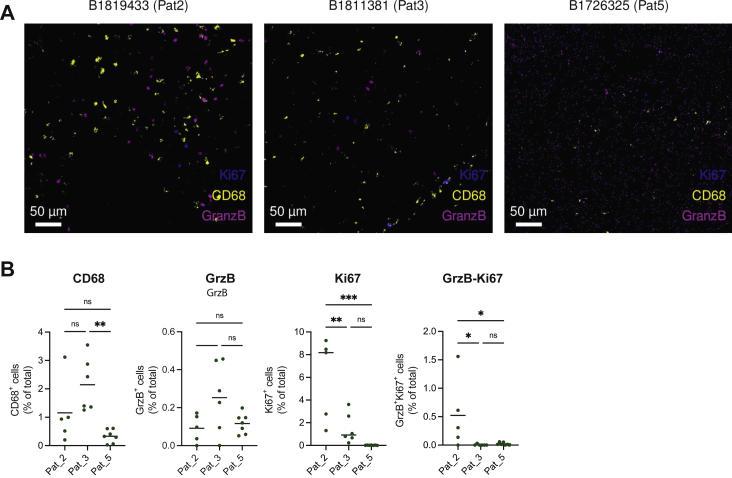
**Visualization of immune cell types in glioblastoma tissue sections of patients after treatment with anti-e**pidermal **growth factor receptor immunoliposomes loaded with doxorubicin (anti-EGFR ILs-dox).** (A) Representative mass cytometric images for patient 2 (left), patient 3 (center), and patient 5 (right) are shown. (B) Quantification of different cell populations including macrophages (CD68 cells), cytotoxic lymphocytes (GranzB), and proliferating cells (Ki67). Frequencies were determined as percentage of total cells. Differences were statistically analyzed by one-way analysis of variance testing. ∗P < 0.05, ∗∗P < 0.01, ∗∗∗P < 0.001. ns, not significant.

To comprehensively quantify immune cell heterogeneity and spatial organization of glioblastoma tissue after anti-EGFR ILs-dox treatment, glioblastoma samples were collected from three patients who were planned for resection of relapsed disease and received anti-EGFR ILs-dox 24 h before surgery. Tissue samples were formalin-fixed and paraffin-embedded at the University Hospital of Basel and processed for IMC. Among the immune cell type included in our IMC panel, macrophages and microglia were the predominant population in each patient tested (Figure 2). CD68^+^ cell frequencies were relatively high in the first two patients treated (Figure 2). However, the third patient who had a prolonged survival showed a clearly reduced number of CD68^+^ macrophages in the specimen and also a lower proliferation of glioma cells (Figure 2). Although the frequencies of other cell populations including B and T cells were assessed using our IMC panel, very few of these immune cells were detected in the samples tested. However, granzyme B-positive cytotoxic cells and proliferating Ki67^+^ and granzyme B double-positive cells were found mainly in patient 2 and 3 but not patient 5 (Figure 2).

## Discussion

We aimed to explore the treatment of patients with EGFR-amplified glioblastoma with EGFR-targeted immunoliposomes containing doxorubicin. An important question was whether immunoliposomes could be used to target malignant gliomas as liposomes generally do not cross the intact blood–brain barrier (BBB). The BBB consists of tightly controlled layers of endothelial and glial cells that block the passage of toxic substances.^24^^,^^25^ However, as soon as brain tumors develop a certain size, tumor neo-vascularization and tumor neo-angiogenesis develop, resulting in a loss of the integrity of the BBB.^26^ Recent experimental evidence strongly suggests that gliomas and in particular glioblastomas can be targeted with nanoparticles.^27^^,^^28^ Although there is preclinical evidence, no trial has shown to date that tumor-targeted liposomes can reach the tumor tissue in glioblastoma patients. Here, we demonstrate that 24 h after the application of the anti-EGFR ILs-dox, a significant concentration of doxorubicin was detectable in the glioblastoma tissue, while anti-EGFR ILs-dox were not able to cross the BBB at clinically relevant levels as the levels found in the CSF were negligible, although high levels of doxorubicin had been detected meanwhile in the plasma. In trials using intravenously injected doxorubicin, similar levels of doxorubicin were observed in liver tissue.^29^, ^30^, ^31^ This is the first time that targeted delivery of immunoliposomes to glioblastoma in patients has been demonstrated. Targeted immunoliposomes could not only be used to transfer cytotoxic drugs but also potent immune-stimulating agents such as cytokines.^32^^,^^33^

This study also evaluated safety data of the application of anti-EGFR ILs-dox in patients with relapsed glioblastoma. In the first-in-human phase I clinical study, anti-EGFR ILs-dox were infused intravenously at escalating doses (doxorubicin 5 mg/m^2^, 10 mg/m^2^, 20 mg/m^2^, 30 mg/m^2^, 40 mg/m^2^, 50 mg/m^2^, and 60 mg/m^2^) once every 4 weeks for a maximum of six cycles in a total of 26 patients.^21^ The primary endpoint was to establish the maximum tolerated dose. Two patients received a dose of 60 mg/m^2^ and had dose-limiting toxicities (one had neutropenia and the other had anemia); therefore, the maximum tolerated dose was defined as 50 mg/m^2^. At 50 mg/m^2^ and at all lower doses, anti-EGFR ILs-dox were well tolerated; grade 1 skin toxicity occurred in two patients, and only 22 serious adverse events were observed in 17 patients, mostly due to tumor progression.^21^ In the present trial, we observed one case of severe pneumonitis due to treatment with anti-EGFR ILs-dox, which has not been reported previously. However, pneumonitis has been reported as side-effect of treatment with liposomal doxorubicin before.^34^, ^35^, ^36^ Application of anti-EGFR ILs-dox before surgery of glioblastoma relapse was feasible and no toxicity or complications occurred in three patients treated in the study. We carried out a multiplex staining to characterize tumor cells and the tumor microenvironment of tumor samples after the treatment with immunoliposomes. Although we found some differences, the numbers of patients are too low to derive any conclusions.

The main limitation of this study is the small sample size with only few patients treated. Although we clearly demonstrate a delivery of immunoliposomes to glioblastoma tissue, no other definitive conclusions can be drawn from this trial. As there was no control group, we cannot exclude that the concentration of doxorubicin in tumor tissue was attained by the systemic level of doxorubicin and thus without the administration of liposomes. Most findings are hypothesis generating, supporting investigation in larger appropriately designed studies.

Taken together, our data suggest that targeted immunoliposomes can be used to deliver cytotoxic and also potentially immune-modulatory molecules to a significant amount to glioblastoma tissue, warranting further clinical evaluation of this approach.

## References

[1] Stupp R., Mason W.P., van den Bent M.J. (2005). Radiotherapy plus concomitant and adjuvant temozolomide for glioblastoma. N Engl J Med.

[2] Wirsching H.G., Galanis E., Weller M. (2016). Glioblastoma. Handb Clin Neurol.

[3] Wen P.Y., Weller M., Lee E.Q. (2020). Glioblastoma in adults: a Society for Neuro-Oncology (SNO) and European Society of Neuro-Oncology (EANO) consensus review on current management and future directions. Neuro Oncol.

[4] Roth P., Hottinger A.F., Hundsberger T. (2020). A contemporary perspective on the diagnosis and treatment of diffuse gliomas in adults. Swiss Med Wkly.

[5] Hottinger A.F., Pacheco P., Stupp R. (2016). Tumor treating fields: a novel treatment modality and its use in brain tumors. Neuro Oncol.

[6] Stupp R., Taillibert S., Kanner A. (2017). Effect of tumor-treating fields plus maintenance temozolomide vs maintenance temozolomide alone on survival in patients with glioblastoma: a randomized clinical trial. J Am Med Assoc.

[7] Weller M., Le Rhun E., Preusser M. (2019). How we treat glioblastoma. ESMO Open.

[8] Giotta Lucifero A., Luzzi S., Brambilla I. (2020). Potential roads for reaching the summit: an overview on target therapies for high-grade gliomas. Acta Biomed.

[9] Taal W., Oosterkamp H.M., Walenkamp A.M. (2014). Single-agent bevacizumab or lomustine versus a combination of bevacizumab plus lomustine in patients with recurrent glioblastoma (BELOB trial): a randomised controlled phase 2 trial. Lancet Oncol.

[10] Wick W., Gorlia T., Bendszus M. (2017). Lomustine and bevacizumab in progressive glioblastoma. N Engl J Med.

[11] Van Den Bent M., Eoli M., Sepulveda J.M. (2020). INTELLANCE 2/EORTC 1410 randomized phase II study of Depatux-M alone and with temozolomide vs temozolomide or lomustine in recurrent EGFR amplified glioblastoma. Neuro Oncol.

[12] Yang J., Yan J., Liu B. (2017). Targeting EGFRvIII for glioblastoma multiforme. Cancer Lett.

[13] Eskilsson E., Rosland G.V., Solecki G. (2018). EGFR heterogeneity and implications for therapeutic intervention in glioblastoma. Neuro Oncol.

[14] Francis J.M., Zhang C.Z., Maire C.L. (2014). EGFR variant heterogeneity in glioblastoma resolved through single-nucleus sequencing. Cancer Discov.

[15] Choi B.D., Maus M.V., June C.H. (2019). Immunotherapy for glioblastoma: adoptive T-cell strategies. Clin Cancer Res.

[16] O'Rourke D.M., Nasrallah M.P., Desai A. (2017). A single dose of peripherally infused EGFRvIII-directed CAR T cells mediates antigen loss and induces adaptive resistance in patients with recurrent glioblastoma. Sci Transl Med.

[17] Mamot C., Drummond D.C., Greiser U. (2003). Epidermal growth factor receptor (EGFR)-targeted immunoliposomes mediate specific and efficient drug delivery to EGFR- and EGFRvIII-overexpressing tumor cells. Cancer Res.

[18] Mamot C., Drummond D.C., Hong K., Kirpotin D.B., Park J.W. (2003). Liposome-based approaches to overcome anticancer drug resistance. Drug Resist Updat.

[19] Wang D., Sun Y., Liu Y. (2018). Clinical translation of immunoliposomes for cancer therapy: recent perspectives. Expert Opin Drug Deliv.

[20] Wicki A., Ritschard R., Loesch U. (2015). Large-scale manufacturing of GMP-compliant anti-EGFR targeted nanocarriers: production of doxorubicin-loaded anti-EGFR-immunoliposomes for a first-in-man clinical trial. Int J Pharm.

[21] Mamot C., Ritschard R., Wicki A. (2012). Tolerability, safety, pharmacokinetics, and efficacy of doxorubicin-loaded anti-EGFR immunoliposomes in advanced solid tumours: a phase 1 dose-escalation study. Lancet Oncol.

[22] Wen P.Y., Macdonald D.R., Reardon D.A. (2010). Updated response assessment criteria for high-grade gliomas: response assessment in neuro-oncology working group. J Clin Oncol.

[23] Capper D., Jones D.T.W., Sill M. (2018). DNA methylation-based classification of central nervous system tumours. Nature.

[24] Daneman R., Prat A. (2015). The blood-brain barrier. Cold Spring Harb Perspect Biol.

[25] Abbott N.J., Ronnback L., Hansson E. (2006). Astrocyte-endothelial interactions at the blood-brain barrier. Nat Rev Neurosci.

[26] Plate K.H., Scholz A., Dumont D.J. (2012). Tumor angiogenesis and anti-angiogenic therapy in malignant gliomas revisited. Acta Neuropathol.

[27] Chou S.T., Patil R., Galstyan A. (2016). Simultaneous blockade of interacting CK2 and EGFR pathways by tumor-targeting nanobioconjugates increases therapeutic efficacy against glioblastoma multiforme. J Control Release.

[28] Karim R., Palazzo C., Evrard B., Piel G. (2016). Nanocarriers for the treatment of glioblastoma multiforme: current state-of-the-art. J Control Release.

[29] Lee Y.T., Chan K.K., Harris P.A., Cohen J.L. (1980). Distribution of adriamycin in cancer patients: tissue uptakes, plasma concentration after IV and hepatic IA administration. Cancer.

[30] Chan K.K., Cohen J.L., Gross J.F. (1978). Prediction of adriamycin disposition in cancer patients using a physiologic, pharmacokinetic model. Cancer Treat Rep.

[31] Benjamin R.S., Riggs C.E., Bachur N.R. (1977). Plasma pharmacokinetics of adriamycin and its metabolites in humans with normal hepatic and renal function. Cancer Res.

[32] Zhang Y., Li N., Suh H., Irvine D.J. (2018). Nanoparticle anchoring targets immune agonists to tumors enabling anti-cancer immunity without systemic toxicity. Nat Commun.

[33] Kwong B., Gai S.A., Elkhader J. (2013). Localized immunotherapy via liposome-anchored Anti-CD137 + IL-2 prevents lethal toxicity and elicits local and systemic antitumor immunity. Cancer Res.

[34] Zhou T., Shen Q., Peng H. (2018). Incidence of interstitial pneumonitis in non-Hodgkin's lymphoma patients receiving immunochemotherapy with pegylated liposomal doxorubicin and rituximab. Ann Hematol.

[35] Meng L., Huang L., Xu Y. (2020). Incidence of interstitial pneumonitis in breast cancer patients treated with pegylated liposomal doxorubicin. Cancer Chemother Pharmacol.

[36] Inaba K., Arimoto T., Hoya M. (2012). Interstitial pneumonitis induced by pegylated liposomal doxorubicin in a patient with recurrent ovarian cancer. Med Oncol.

