# Supplementary material for: Targeting immunoliposomes to EGFR-positive glioblastoma
Source: ESMO Open. 2022 Jan 5;7(1):100365. doi: 10.1016/j.esmoop.2021.100365 (PMC8741448; doi:10.1016/j.esmoop.2021.100365)
Supplement: Supplement [file mmc1.pdf]

A pharmacokinetic phase 1 study of anti-EGFR-immunoliposomes loaded with doxorubicin in patients with relapsed or refractory high-grade gliomas

Clinical Study Protocol

GBM-LIPO Study

Study Type:	Clinical trial with Investigational Medicinal Product (IMP)
Study Categorisation:	Risk category C according to LHR
Study Registration:	clinicaltrial.gov NCT03603379
Study Identifier:	GBM-LIPO
Sponsor	University Hospital Basel (Sponsor)
Principal Investigator:	Dr Heinz Läubli (Principal investigator)<br>Dep. Oncology<br>University Hospital Basel<br>Petersgraben 4, 4031 Basel<br>heinz.laeubli@usb.ch
Investigational Product:	C225-ILs-dox
Protocol Version and Date:	Version 1.1 06.08.2018

Signature Page(s)

Study Title	A pharmacokinetic phase 1 study of anti-EGFR-immunoliposomes loaded with doxorubicin in patients with relapsed or refractory high-grade gliomas

The Sponsor-Investigator and trial statistician have approved the protocol version [1.0 (dated 06.04.2018], and confirm hereby to conduct the study according to the protocol, current version of the World Medical Association Declaration of Helsinki, ICH-GCP guidelines or ISO 14155 norm if applicable and the local legally applicable requirements.

Sponsor-Investigator:
Dr. Heinz Läubli

Basel, 06.08.2018

Place/Date Signature

Data analysis, statistical considerations:
PD Dr. Benjamin Kasenda

Basel, 08.08.2018

Place/Date Signature

Local Principal Investigators at study sites*:

I have read and understood this trial protocol and agree to conduct the trial as set out in this study protocol, the current version of the World Medical Association Declaration of Helsinki, ICH-GCP guidelines or ISO 14155 norm and the local legally applicable requirements.

Site	Universitätsspital Basel
Principal investigator	Dr. med. Heinz Läubli

Basel, 06.08.2018

Place/Date Signature

Local Principal Investigators at study sites*:

I have read and understood this trial protocol and agree to conduct the trial as set out in this study protocol, the current version of the World Medical Association Declaration of Helsinki, ICH-GCP guidelines or ISO 14155 norm and the local legally applicable requirements.

Site	Kantonsspital Aarau
Principal investigator	Prof. Dr. med. Christoph Mamot

Aarau, 07.08.2018

Place/Date Signature

Table of Contents

STUDY SYNOPSIS **8**
STUDY SUMMARY IN LOCAL LANGUAGE **12**
ABBREVIATIONS **13**
STUDY SCHEDULE **14**
1. STUDY ADMINISTRATIVE STRUCTURE **15**
1.1 Sponsor, Sponsor-Investigator 15
1.2 Principal Investigator(s) 15
1.3 Statistician ("Biostatistician") 15
1.4 Laboratory 15
1.5 Monitoring institution 15
1.6 Data Safety Monitoring Committee 15
1.7 Any other relevant Committee, Person, Organisation, Institution 15
2. ETHICAL AND REGULATORY ASPECTS **16**
2.1 Study registration 16
2.2 Categorisation of study 16
2.3 Competent Ethics Committee (CEC) 16
2.4 Competent Authorities (CA) 16
2.5 Ethical Conduct of the Study 16
2.6 Declaration of interest 16
2.7 Patient Information and Informed Consent 16
2.8 Participant privacy and confidentiality 17
2.9 Early termination of the study 17
2.10 Protocol amendments 17
2.10.1 Substantial Amendment 17
2.10.2 Safety Amendment 17
2.10.3 Non-substantial amendment 17
2.11 Trial Activation 17
2.12 Study duration and schedule 18
3. BACKGROUND AND RATIONALE **18**
3.1 Background and Rationale 18
3.2 Investigational Product and Indication 18
3.3 Preclinical Evidence 19
3.4 Clinical Evidence to Date 19
3.5 Rationale for the intended purpose in study 19
3.6 Explanation for choice of comparator (or placebo) 19
3.7 Risks / Benefits 19
3.8 Justification of choice of study population 19
4. STUDY OBJECTIVES **20**
4.1 Overall Objective 20
4.2 Primary Objective 20
4.3 Secondary Objectives 20
4.4 Safety Objectives 20

5. **STUDY OUTCOMES** **20**
5.1 Primary Outcome 20
5.1.1 Measurement of pharmacokinetics 20
5.2 Secondary Outcomes 21
5.3 Other Outcomes of Interest 21
5.4 Safety Outcomes 21
6. **STUDY DESIGN** **21**
6.1 General study design and justification of design 21
6.2 Methods of minimising bias 22
7. **STUDY POPULATION** **22**
7.1 Eligibility criteria 22
7.1.1 Inclusion criteria 22
7.1.2 Exclusion criteria 22
7.2 Recruitment and screening 23
7.3 Assignment to study groups 23
7.4 Criteria for withdrawal / discontinuation of participants 23
8. **STUDY INTERVENTION** **23**
8.1 Identity of Investigational Products 23
8.1.1 Experimental Intervention 23
8.1.2 Control Intervention 23
8.1.3 Packaging, Labelling and Supply (re-supply) 23
8.2 Product quality and complaint handling 24
8.2.1 Storage Conditions 24
8.3 Administration of experimental intervention 24
8.3.1 Schedule 24
8.3.2 Dose calculation and administration 24
8.3.3 Premedication or supportive medication 24
8.3.4 Warnings and precautions 24
8.3.5 Control Intervention 24
8.4 Dose modifications 24
8.4.1 Hand-foot syndrome 25
8.4.2 Mucositis/stomatitis 25
8.4.3 Haematological toxicity 25
8.4.4 Infection 26
8.4.5 Cardiotoxicity (decrease of LVEF) 26
8.4.6 Bilirubin 26
8.4.7 Other adverse events 26
8.5 Compliance with study intervention 27
8.6 Data Collection and Follow-up for withdrawn participants 27
8.7 Trial specific preventive measures 27
8.8 Concomitant Interventions (treatments) 27
8.9 Study Drug Accountability 27
8.10 Return or Destruction of Study Drug 27

9. STUDY ASSESSMENTS 27
9.1 Study flow chart 28
10. REGISTRATION 28
After patient registration 28
11. ASSESSMENTS OF OUTCOMES 28
11.1 Assessment of primary outcome 28
11.2 Assessment of secondary outcomes 28
11.3 Assessment of other outcomes of interest 29
11.4 Assessment of safety outcomes 29
11.4.1 Adverse events 29
11.4.2 Laboratory parameters 29
11.4.3 Vital signs 29
11.4.4 End of treatment visit 29
11.4.5 Evaluation in follow-up phase 29
11.5 Assessments in participants who prematurely stop the study 29
11.6 Procedures at each visit 29
11.6.1 To be performed before registration (Screening): 29
11.6.2 Can be performed within 28 days before registration (Screening, day -28 to -1): 29
11.6.3 To be performed within 7 days before registration (Screening, day -7 to -1): 29
11.6.4 To be performed at each treatment visit (every 4 weeks, treatment phase ± 5 days) 30
11.6.5 Lumbar puncture after 1st treatment application 30
11.6.6 Surgical resection of relapsed tumour 30
11.6.7 To be performed at the end of treatment visit (28 days after last treatment application ± 5 days) 30
11.6.8 To be performed at each visit during follow-up phase (every 6 weeks ± 5 days for 12 months) 30
11.6.9 To be performed at the end of treatment visit (8 weeks after last follow-up visit) 30
12. SAFETY 30
12.1 Drug studies 30
12.1.1 Definition and assessment of (serious) adverse events and other safety related events 31
12.1.2 Reporting of serious adverse events (SAE) and other safety related events 32
12.1.3 Reporting of SAEs and SUSARs by the Sponsor 32
12.1.4 Periodic reporting on safety to principal investigators 33
12.1.5 Reporting and Handling of Pregnancies 33
12.1.6 Follow up of (Serious) Adverse Events 33
12.1.7 Pregnancy 33
13. STATISTICAL METHODS 33
13.1 Hypothesis 33
13.2 Determination of Sample Size 33
13.3 Statistical criteria of termination of trial 33
13.4 Planned Analyses 33
13.4.1 Datasets to be analysed, analysis populations 33
13.4.2 Primary Analysis 33
13.4.3 Secondary Analyses 34
13.4.4 Interim analyses 34

13.4.5 Safety analysis 34
13.4.6 Deviation(s) from the original statistical plan 34
13.5 Handling of missing data and drop-outs 34
14. QUALITY ASSURANCE AND CONTROL 34
14.1 Data handling and record keeping / archiving 35
14.1.1 Investigator's file 35
14.1.2 Useful tools ..... **Fehler! Textmarke nicht definiert.**
14.1.3 Record retention 35
14.1.4 Case Report Forms 35
14.1.5 Specification of source documents 35
14.1.6 Record keeping / archiving 36
14.2 Data management 36
14.2.1 Data Management System 36
14.2.2 Data security, access and back-up 36
14.2.3 Analysis and archiving 36
14.2.4 Electronic and central data validation 36
14.3 Monitoring 36
14.4 Audits and Inspections 36
14.5 Confidentiality, Data Protection 36
14.6 Storage of biological material and related health data 36
15. PUBLICATION AND DISSEMINATION POLICY 36
16. FUNDING AND SUPPORT 37
16.1 Funding 37
16.2 Other Support 37
17. INSURANCE 37
18. REFERENCES 38
19. APPENDICES 40

STUDY SYNOPSIS

**Sponsor / Sponsor-Investigator**	University Hospital Basel / Dr. Heinz Läubli
**Study Title:**	A pharmacokinetic phase 1 study of anti-EGFR-immunoliposomes loaded with doxorubicin in patients with relapsed or refractory high-grade gliomas
**Short Title / Study ID:**	C225-ILs-dox in high-grade gliomas
**Protocol Version and Date:**	Version 1.1,06.08.2018
**Trial registration:**	Study to be registered at clinicaltrials.gov and the Swiss National Clinical Trials Portal
**Study category and Rationale**	The doxorubicin-loaded anti-EGFR-immunoliposomes (C225-ILs-dox) is a medication without marketing authorization in Switzerland/EU. According to the Swiss HRA and its corresponding Ordinance KlinV/Oclin on clinical trials, this trial is classified as category C.
**Clinical Phase:**	Phase I pharmacokinetic study (not first in man study)
**Background and Rationale:**	High-grade gliomas have a dismal short-term prognosis with more than 50% of patients in progression 6 months after standard first-line radio-chemotherapy with temozolomide. There are no approved drug-therapy options proven to improve survival after failure of first-line treatment. There is a great medical need for new and effective treatment options to improve prognosis for these patients.<br>Data from a phase I trial (26 patients with different solid EGFR positive tumours) show little toxicity and impressive signs of efficacy of C225-ILs-dox (1). Approximately 40-50% of high-grade gliomas have an EGFR amplification with high EGFR expression (especially glioblastomas), providing a very good biological rationale to test C225-ILs-dox in patients with relapsed high-grade gliomas. However, up to know, there is no clinical evidence, that C225-ILs-dox given intravenously achieve sufficient drug concentration in the CSF. We therefore propose this phase I pharmacokinetic study in patients with relapsed/refractory high-grade gliomas.
**Objective(s):**	To determine the pharmacokinetics of C225-ILs-dox in the CNS during salvage therapy in patients with relapsed/refractory high-grade gliomas.

**Outcome(s):**	Regarding the main endpoint (pharmacokinetics), at each time point (24 hours after first application and day 80) we will calculate:<br>• the ratio of the CSF concentration of doxorubicin (μg/ml) over the total plasma concentration of doxorubicin (μg/ml) [$CSF_{dox}/Plasma_{dox}$]<br>• the ratio of the CSF concentration of the liposomal fraction (μg/ml) over the total plasma concentration of the liposomal fraction (μg/ml) [$CSF_{lipo}/Plasma_{lipo}$]<br>• the ratio of the CSF concentration of the cetuximab-Fab′ (ng/ml) over the total plasma concentration of the cetuximab-Fab′ (ng/ml) [$CSF_{FAB}/Plasma_{FAB}$]<br>Secondary endpoints:<br>• Tumour response according to RANO criteria (2) on the final MRI scan after completion of 4 treatment cycles (cycle 4, day 28).<br>• Best achieved tumour response (1st or second MRI scan) during treatment phase according to RANO criteria (2).<br>• Event free survival, defined as the time between registration to progression, termination of therapy for toxicity, or death whichever occurs first.<br>• Progression free survival, defined as the time between registration to progression or death whichever occurs first.<br>• Overall survival, defined as the time between registration to death due to any cause.<br>• Toxicity as graded by the CTCAE Version 4.0.
**Study design:**	Prospective, single-stage, single-arm, open-label pharmacokinetic study at two Swiss centres (University Hospital Basel, Kantonsspital Aarau)
**Inclusion / Exclusion criteria:**	Main Inclusion criteria:<br>• Glioblastoma (WHO IV)<br>• EGFR amplification (considered amplified if the value is 0.15 above the average signal of chromosome 7)<br>• Measurable or evaluable disease<br>• At least one prior line of therapy<br>• ECOG PS of 0-2<br>• Patient ≥ 18 years<br>Main Exclusion criteria:<br>• Contraindications for lumbar puncture<br>• Previous therapy with more than 240 mg/m^2 of doxorubicin or more than 450 mg/m^2 of epirubicin for any indication<br>• Life expectancy less than 2 months<br>• Breastfeeding or pregnancy<br>• Any other serious underlying medical, psychiatric, psychological, familial or geographical condition, which in the judgment of the investigator may interfere with the planned staging, treatment and follow-up, affect patient compliance or place the patient at high risk from treatment-related complications.

**Measurements and procedures:**	All patients will be discussed at an interdisciplinary neuro-oncology tumour board at diagnosis of relapse. Irrespective of eligibility for resection of relapsed disease, patients will be evaluated for study eligibility. We aim to include at least 3 patients with resectable disease to assess anti-EGFR-IL-dox concentration in the primary brain tumour.<br>24 hours after the first intravenous application of anti-EGFR-IL-dox, patients with resectable tumour will undergo resection and have PK samples taken from PB and CSF (during operation). For those patients with relapsed tumours, but deemed unresectable, 24 hours after first application of anti-EGFR-IL-dox, PK samples from peripheral blood (venous puncture) and CSF (by lumbar puncture) will be taken. All PK samples (PB, CSF and resected tumour tissue) will be assessed for the presence of anti-EGFR-IL-dox. There will be one MRI brain scan for interim assessment after the 2nd cycle (day 14, cycle 2). After the final MRI brain scan (day 28, cycle 4), further imaging is at the discretion of the treating physician. Afterwards, patients enter the follow-up phase (until disease progression or at maximum for 12 months). We will collect safety data until 3 months after last application.
**Study Product / Intervention:**	C225-ILs-dox will be administered at a dose of 50 mg/m2. i.v., on day 1 of each cycle, cycle length is 28 days. In total, 4 cycles are planned to be applied.
**Control Intervention (if applicable):**	N/A
**Number of Participants with Rationale:**	For this pharmacokinetic study, we have chosen a convenience sample size of 9 patients. Therefore, no power calculation was conducted.
**Study Duration:**	Estimated accrual duration: 12-18 months<br>Duration of trial therapy (per patient): 3 months<br>Duration of follow-up (per patient): 12 months<br>Estimated trial duration in total: 36 months (3 years)<br>The trial may be stopped early if new scientific data become available which change the assessment of risk/benefit.
**Study Schedule:**	11/2018 First-Participant-In (planned)<br>11/2021 Last-Participant-Out (planned)
**Investigator(s):**	Dr. Heinz Läubli (Basel) Prof. Dr. Christoph Mamot (Aarau)
**Study Centre(s):**	This trial will be conducted at 2 sites (Basel, Aarau)

**Statistical Considerations:**	**Sample size**<br>No sample size calculation was done for this exploratory pharmacokinetic study; In order to faithfully predict the bioavilability of C225-ILs-dox and the concentration of active compounds in the CNS, 9 patients will be treated. We aim to collect of 3 patients resection material (patients undergoing surgery at the timepoint of relapse).<br>**Statistical analyses**<br>We will describe the patient population baseline characteristics including previous treatments and time in remission after last treatment using descriptive statistics with frequencies (proportions) and means/medians (range) as appropriate.<br>We will illustrate the ratios of concentration using plots showing the median/mean and range at each time point. Plots for CSF and peripheral blood concentrations will also be created separately. We will calculate the number (proportion) of patients in which no C225-ILs-dox concentration was detected in the CSFf.<br>Regarding tumour response, we will calculate frequency (proportions with all patients registered in the denominator) of tumour response status as per RANO criteria: responders (complete response or partial response), non-responder (stable disease, progressive disease). Patients without any response assessment due to any cause will be considered as non-responders. Regarding time-to-event data, we will use the Kaplan-Meier product estimate to illustrate survival probabilities for EFS and OS. Toxicity events per patient will be summarized using descriptive statistics (frequencies, proportions).
**GCP Statement:**	This study will be conducted in compliance with the protocol, the current version of the Declaration of Helsinki, the ICH-GCP or ISO EN 14155 (as far as applicable) as well as all national legal and regulatory requirements.

STUDY SUMMARY IN LOCAL LANGUAGE

Die Studie untersucht die Anwendung von einem Medikament mit dem Namen C225-ILs-dox (anti-EGFR-Immunliposomen). Dieses Medikament besteht aus zwei Bestandteilen, nämlich einer Chemotherapie (Doxorubicin) und einem Antikörper (Cetuximab). Die Studie möchte untersuchen, ob C225-ILs-dox nach Gabe über die Vene im Hirngewebe und der Hirn-/Rückenmarksflüssigkeit ankommt. Zudem wird die Verträglichkeit und Wirksamkeit dieses Medikamentes geprüft.

Es können alle erwachsenen Personen teilnehmen, die an einem erneut gewachsenen Glioblastom leiden (Rückfall des Glioblastoms). Des Weiteren muss eine sogenannte EGFR Amplifikation (spezifische genetische Veränderung des Tumors, welche in der Gewebsprobe bei Erstdiagnose bestimmt wird, EGFR Amplifikation) vorliegen.

Die Behandlung mit dem Medikament dauert maximal 16 Wochen (4 x 4 Wochen pro Zyklus). Falls der Tumor trotz der Behandlung wachsen sollte oder Nebenwirkungen auftreten, wird die Behandlung vorher abgebrochen.

ABBREVIATIONS

Provide a list of abbreviations used on the protocol - to be completed

AE	Adverse Event
CA	Competent Authority (e.g. Swissmedic)
CEC	Competent Ethics Committee
CRF	Case Report Form
ClinO	Ordinance on Clinical Trials in Human Research *(in German: KlinV, in French: OClin)*
eCRF	Electronic Case Report Form
CTCAE	Common terminology criteria for adverse events
DSUR	Development safety update report
EGFR	Epithelial growth factor receptor
GCP	Good Clinical Practice
IB	Investigator's Brochure
Ho	Null hypothesis
H1	Alternative hypothesis
HFG	Humanforschungsgesetz (Law on human research)
HMG	Heilmittelgesetz
HRA	Federal Act on Research involving Human Beings
IMP	Investigational Medicinal Product
IIT	Investigator-initiated Trial
ISO	International Organisation for Standardisation
ITT	Intention to treat
KlinV	Verordnung über klinische Versuche in der Humanforschung *(in English: ClinO, in French OClin)*
LPTh	Loi sur les produits thérapeutiques
LRH	Loi fédérale relative à la recherche sur l'être humain
MD	Medical Device
OClin	Ordonnance sur les essais cliniques dans le cadre de la recherche sur l'être humain *(in German : KlinV, in English : ClinO)*
PI	Principal Investigator
SDV	Source Data Verification
SOP	Standard Operating Procedure
SPC	Summary of product characteristics
SUSAR	Suspected Unexpected Serious Adverse Reaction
TMF	Trial Master File

STUDY SCHEDULE

Investigations	Screening	Treatment phase							Follow-up
Visit	1	2	3	4	5	6	7	8	
Day	-28 to -1	0	1	28 (± 5 days)	42 (± 5 days)	52 (± 5 days)	80 (± 5 days)	104 (± 5 days)	Starting 28 after day 1 of last treatment cycle (every 6 weeks)
Informed Consent	X								
Demographics	X								
Medical History	X								
In- /Exclusion Criteria	X								
Physical Examination	X	X		X		X	X		X
Pregnancy Test	X								
EGFR amplification test	X								
ECHO, ECG	X								
proBNP	X					X			X
Vital Signs	X^{+}	X		X		X	X		
ECOG	X^{+}	X		X		X	X		X
Height/Weight	X^{+}								
Full blood count	X^{+}								X
Liver function tests	X^{+}	X		X		X	X		X
Kidney function test	X^{+}	X		X		X	X		X
Clotting tests			X						
Study Medication		X		X		X	X		
PK samples (CSF/Operation)			X*						

PK samples peripheral blood			X*	X		X	X	X	
MRI brain	X				X			X	
Adverse Events	X	X	X	X	X	X	X	X	X

* 24 hours after application of 1st treatment; + if conducted within 3 days before first treatment not necessary to repeat

1. STUDY ADMINISTRATIVE STRUCTURE

Protocol writing committee:

Steering committee: Heinz Läubli, Benjamin Kasenda, and Christoph Mamot, Andreas Wicki

1.1 Sponsor, Sponsor-Investigator

University Hospital Basel represented by Dr. med Heinz Läubli

1.2 Principal Investigator(s)

Dr. med Heinz Läubli, University Hospital Basel, Department of Medical Oncology
heinz.laeubli@usb.ch
Prof. Dr. med Christopf Mamot, Kantonsspital Aarau, Department of Medical Oncology
christoph.mamot@ksa.ch.

1.3 Statistician ("Biostatistician")

N/A

1.4 Laboratory

N/A

1.5 Monitoring institution

Clinical Trials Unit Basel University Hospital

1.6 Data Safety Monitoring Committee

N/A

1.7 Any other relevant Committee, Person, Organisation, Institution

N/A

2. ETHICAL AND REGULATORY ASPECTS

This protocol was written and the trial will be carried out in accordance with the principles enunciated in the current version of the Declaration of Helsinki, the guidelines of Good Clinical Practice (GCP) issued by ICH, the applicable Swiss HRA and its associated Ordinances and the requirements from the Swiss regulatory bodies. The protocol, the patient information and consent form, as well as all other trial-related documents shall be submitted to all involved ECs and to the competent authority in agreement with local legal requirements for formal authorization. Any amendment to the protocol or patient information and consent form will be submitted for authorization to these institutions. The decision of the ECs and competent authority with regard to the conduct of the trial will be made in written to the Sponsor prior to trial initiation. Any substantial amendment to the protocol (except for safety reasons) can only be implemented at a site after obtaining written authorization by the corresponding regulatory bodies. Patient recruitment can only take place after the site has officially been opened for accrual by the Sponsor. Sites have to adhere to the Swiss HRA and all applicable local regulatory guidelines.

2.1 Study registration

The study will be registered on clinicaltrials.gov (NCTXXXX) and in the Swiss Federal Complementary Database (Portal).

2.2 Categorisation of study

Clinical trial with Investigational Medicine Product (IMP). The doxorubicin-loaded anti-EGFR-immunoliposomes is a medication without marketing authorization in Switzerland/EU. According to the Swiss HRA and its corresponding Ordinance KlinV/Oclin on clinical trials, this trial is classified as category C.

2.3 Competent Ethics Committee (CEC)

The responsible investigator at each site ensures that approval from an appropriately constituted Competent Ethics Committee (CEC) is sought for the clinical study.

The responsible investigator at each site will follow the clinical trial protocol. No changes will be made to the protocol without prior Sponsor and CEC approval, except where necessary to eliminate apparent immediate hazards to study participants.

Premature study end or interruption of the study will be reported within 15 days. The regular end of the study will be reported to the CEC within 90 days, the final study report shall be submitted within one year after study end. Amendments are reported according to chapter 2.10.

2.4 Competent Authorities (CA)

The Sponsor will obtain approval from the competent authority (Swissmedic) before the start of the clinical trial. The sponsor will report any premature study discontinuation to the CA within 30 days. In case a study termination as planned, the final report will be submitted to the CA within 3 months after official closure of the study.

2.5 Ethical Conduct of the Study

The study will be carried out in accordance to the protocol and with principles enunciated in the current version of the Declaration of Helsinki, the guidelines of Good Clinical Practice (GCP) issued by ICH, in case of medical device: the European Directive on medical devices 93/42/EEC and the ISO Norm 14155 and ISO 14971, the Swiss Law and Swiss regulatory authority's requirements. The CEC and regulatory authorities will receive annual safety and interim reports and be informed about study stop/end in agreement with local requirements.

2.6 Declaration of interest

The involved members of the protocol writing committee and steering committee do not have any conflicts of interest with regard to design, set-up and conduct of this study.

2.7 Patient Information and Informed Consent

The informed consent procedure must conform to the Swiss law and the guidelines on GCP issued by ICH. All patients will be informed of the aims and procedures of the trial, the possible AEs, how to react in case an AE occurs, and possible hazards to which they will be exposed. They will be informed as to the strict confidentiality of their patient data, but they need to know that their medical records may be reviewed for trial purposes by authorized individuals other than their treating physician. The investigator must provide the patient with sufficient opportunity to consider whether or not to participate and minimize the possibility of coercion or undue influence. The information provided shall be in a language intelligible

to the patient and may not include any content that appears to waive any of the patient's legal rights, or appears to release the investigator, the sponsor, or the institution from liability for negligence.

It will be emphasized that participation is voluntary and that the patient is allowed to refuse further participation in the trial whenever he/she wants. This will not prejudice the patient's subsequent care. Informed consent will be obtained before registration and prior to any protocol-specific procedures. Informed consent shall be obtained on a written form approved by the local EC and signed and personally dated by the patient and the investigator. The patient information as well as a copy or original of the signed and dated informed consent will be handed to the patient. No inclusion of legally incompetent patients is permitted in this trial.

In case new results become available that shift the risk/benefit ratio, the patient should re-consent. Patients refusing to accept non-mandatory translational projects can nevertheless participate in the trial. The patient has at least 24 hours to decide if he wants to participate in the trial and also participate in the translational analysis.

2.8 Participant privacy and confidentiality

The investigator affirms and upholds the principle of the participant's right to privacy and that they shall comply with applicable privacy laws. Especially, anonymity of the participants shall be guaranteed when presenting the data at scientific meetings or publishing them in scientific journals.

Individual subject medical information obtained as a result of this study is considered confidential and disclosure to third parties is prohibited. Subject confidentiality will be further ensured by utilising subject identification code numbers to correspond to treatment data in the computer files.

For data verification purposes, authorised representatives of the Sponsor (-Investigator), a competent authority (e.g. Swissmedic), or an ethics committee may require direct access to parts of the medical records relevant to the study, including participants' medical history.

2.9 Early termination of the study

The Sponsor-Investigator may terminate the study prematurely according to certain circumstances, for example:

- ethical concerns,
- insufficient participant recruitment,
- when the safety of the participants is doubtful or at risk, respectively,
- alterations in accepted clinical practice that make the continuation of a clinical trial unwise,
- early evidence harm of the experimental intervention

2.10 Protocol amendments

2.10.1 Substantial Amendment

Any amendment which may have an impact on the conduct of the trial, the potential benefit of the trial, or may affect patient safety, including changes of trial objectives, trial design, patient population, sample sizes, trial procedures, or significant administrative aspects. Such an amendment must be accepted by the Steering committee and must have the authorization of the local EC and competent authority (if applicable) prior to implementation.

2.10.2 Safety Amendment

A safety amendment is a special kind of substantial amendment which is released when it is necessary to eliminate immediate hazards to trial participants. A safety amendment requires immediate implementation at local sites and is submitted in parallel for authorization to the ECs and the competent authority (if applicable).

2.10.3 Non-substantial amendment

Non-substantial amendments such as minor corrections and/or clarifications that have no effect on the way the trial is conducted have to be submitted to the ECs once a year, together with the submission of the annual safety report. Non-substantial amendments which affect the evaluation of the competent authority have to be submitted to the CA as soon as possible.

2.11 Trial Activation

The procedure for trial activation at a site is described in the final protocol letter, which is sent to all sites that committed to participate in the trial. All participating sites must follow the instructions given in this letter for the preparation of site documents. Upon receipt of the site documents the Sponsor will submit them to the involved ECs and Swissmedic.

The investigator will only be allowed to register patients into the trial after the ECs and Swissmedic have authorized the trial at the site and the Sponsor has opened the site for accrual.

2.12 Study duration and schedule

Estimated accrual duration: 12-18 months

Duration of trial therapy (per patient): 3 months

Duration of follow-up (per patient): 12 months

Estimated trial duration in total: 36 months (3 years)

The trial may be stopped early if new scientific data become available which change the assessment of risk/benefit.

11/2018 First-Participant-In (planned)

11/2021 Last-Participant-Out (planned)

3. BACKGROUND AND RATIONALE

3.1 Background and Rationale

High-grade malignant gliomas (anaplastic gliomas and glioblastomas) are primary brain tumours with a very poor prognosis (3).The yearly incidence is ~3–5/100'000 with a slight predominance in males. These tumours may develop at all ages, with the peak incidence being in the fifth and sixth decades of life (4).

The current treatment options for patients with newly diagnosed disease include maximal possible resection followed by adjuvant radio-chemotherapy with 60 Gy and the alkylating agent temozolomide which is associated with a survival rate of about 27% after 2 years (median survival 15 months) (5,6). Recently, the addition of tumour treating fields after completion of radio-chemotherapy has led to a slight further prolongation of survival rates (7). However, the great majority of patients with high-grade malignant gliomas relapse 12-15 months after initiation of first-line therapy (5) and treatment options in this situation are very limited (8). Anti-angiogenic therapy with bevacizumab is an approved treatment modality in the relapse setting leading to disease stabilization and tumour regression in some patients, but associated life expectancy after disease progression is still only a few months (9–11). Certainly, there is a great medical need for improving the prognosis for patients with high-grade malignant gliomas.

Among the most frequently found genetic aberrations in high-grade gliomas (especially glioblastoma) is the amplification of the epithelial growth factor receptor (EGFR), which can be detected in about 40-50% of glioblastomas (12). Amplification of EGFR is often accompanied by the appearance of a variant form of EGFR called variant III (EGFRvIII) (13,14). However, targeting EGFR in glioblastoma patients with tyrosine kinases used for the treatment of e.g. lung cancer, is not very attractive, since most of the sensitizing mutations are usually absent (14). However, the frequent presence of EGFR makes it a very interesting molecule for targeting active substances to the tumour microenvironment. EGFR in glioblastoma has been targeted by antibody drug conjugates including ABT-414, which is an antibody targeting EGFR and EGFRvIII coupled to the anti-microtubule agent monomethylauristatin F (15). Initial data is promising confirming the approach of using EGFR-targeting to deploy higher doses of chemotherapy, but improvements and further investigations are warranted.

3.2 Investigational Product and Indication

For preclinical studies, C225-ILs-dox were constructed by using Fab' fragments of the chimeric MAb cetuximab (C225, cetuximab, erbitux®, Merck), which were covalently conjugated to the liposome membrane. This approach was designed to provide maximal drug delivery to cancer cells via a receptor-targeted and internalizing drug carrier that is stable, non-immunogenic, long-lived with extended blood and tissue residence times, and capable of delivering large payloads of diverse types of drugs. In parallel with MAb fragment optimization, conjugation methodology was also optimized. We developed a new micellar incorporation method involving 2-step conjugation of MAb fragments to preformed drug loaded liposomes. First, MAb fragments (Fab') were covalently conjugated to derivatized PEG-phosphatidyl-ethanolamine (MAL-PEG-DSPE) linkers in solution, resulting in immunoconjugates prone to spontaneous micelle formation. Next, the conjugates were incorporated into drug-preloaded liposomes by controlled heating, resulting in MAb fragments covalently conjugated to the termini of PEG chains and anchored to the liposome (1). For this trial, we will use anti-EGFR-IL-dox, thus anti-EGFR Fab' will be conjugated with doxorubicin- containing liposomes (caelyx®) as previously used for the phase I trial (16). The investigational product will be manufactured in our Hospital Pharmacy at the University of Basel under Good Manufacturing Practice (GMP) conditions as it was done for the phase I and the

ongoing phase II trial with anti-EGFR-IL-dox (NCT00445406).

3.3 Preclinical Evidence

The in vitro activity of immun-liposomes containing various reporters or drugs were investigated by Momat et al on EGFR/EGFRvIII-overexpressing cell lines. Flow cytometry and fluorescence microscopy showed that EGFR-targeted immun-liposomes, but not non- targeted liposomes or irrelevant immun-liposomes, were efficiently bound and internalized by EGFR-overexpressing cells, including glioma cells, carcinoma cells, and EGFRvIII stable transfectants. Furthermore, EGFR-targeted immun-liposomes did not bind to non-EGFR-overexpressing cells immun-liposomes. Immun-liposomes were used to deliver cytotoxic drugs doxorubicin, vinorelbine, or methotrexate to EGFR/ EGFRvIII-overexpressing target cells in vitro. In each case, the immun-liposomes agent was significantly more cytotoxic than the corresponding nontargeted liposomal drug in target cells, whereas it was equivalent in cells lacking EGFR/EGFR- vIII overexpression. Therefore, the investigators concluded that EGFR-targeted immun-liposomes provide efficient and targeted delivery of anticancer drugs in cells overexpressing EGFR or EGFRvIII (17).

3.4 Clinical Evidence to Date

Mamot and colleagues have investigated C225-ILs-dox regarding dose limiting toxicities, safety and preliminary efficacy in a phase I dose-escalating study (single centre) including 29 patients with EGFR-overexpressing advanced solid tumours no longer amenable to standard treatment (16). The maximum tolerated dose was defined as 50 mg/m2. Best response to treatment included one complete response, one partial response, and ten stable disease lasting 2 to 12 months. Based on this study, a multi-centre single arm phase II study for women with advanced EGFR expressing breast cancer was initiated and is currently recruiting (NCT00445406).

3.5 Rationale for the intended purpose in study

Because of the chemical construct of the IMP, the route of administration is intravenous. The dose of $50mg/m^2$ and schedule of application is based on a pharmacokinetic dose-escalation study (16).

3.6 Explanation for choice of comparator (or placebo)

N/A

3.7 Risks / Benefits

Patients with relapsed/refractory glioblastoma have a very poor prognosis with only several months remaining survival time. There is no approved medical intervention proven to prolong survival after failure of first-line combined radio-chemotherapy. Therefore, there is a great medical need to explore new treatment options for these patients to subsequently improve prognosis. The associated risks for patients within this study are the potential side effects of the IMP and the potential complications of one lumbar puncture conducted to measure the IMP concentration in the CSF. For patients who have resectable relapsed tumours, the resection would also be conducted outside a clinical study, therefore a potential operation to resect the relapsed tumour is not considered a study related procedure putting the patient at additional risk compared to usual care.

The safety profile of the IMP has been investigated in a previous study of patients with advanced solid tumours (EGFR positive) who have been heavily pre-treated. This study has shown an acceptable safety profile and some promising efficacy signals (16). For the study described herein, we will only include patients with EGFR amplification. By this, we select the group of glioblastoma patients who have, at least based on biological rationale and comparable examples in other tumour types, the highest chance for a clinical meaningful tumour response. The principle of targeting specific characteristics of tumour cells to deliver cytotoxic agents has been shown to be an effective strategy in several other tumours.

Patients will be closely monitored during the treatment phase by experienced clinical teams to minize the risk for treatment associated side effects.

In summary, we believe that the potential benefits regarding tumour control outweigh potential side effects and risks. In addition, results from this study can provide very valuable insights for future studies investigating the principle of EGFR targeted treatment approaches in glioblastoma.

3.8 Justification of choice of study population

Patients with relapsed/refractor glioblastoma have a dismal prognosis with very limited treatment option. The only approved second line treatment after combined radio-chemotherapy is the anti-VEGF antibody bevacizumab, however, there is no evidence for a survival benefit of bevacizumab and the associated clinical benefit lasts only for some weeks. Therefore, there is a great medical need to improve prognosis for these patients. The frequently observed EGFR amplifications in glioblastoma provides the rationale

to investigate drugs targeting this alteration very similar to other targeted agents or drug conjugates in oncology. We therefore plan to investigate C225-ILs-dox in this population of glioblastoma patients.

4. STUDY OBJECTIVES

4.1 Overall Objective

The overall purpose of the study is to investigate the pharmacokinetics (tumour tissues, CSF and peripheral blood) and pre-liminary efficacy of C225-ILs-dox in patients with relapsed/refractory glioblastoma.

4.2 Primary Objective

To investigate CNS penetration of C225-ILs-dox in patients with relapsed EGFR amplified glioblastoma measured in the CSF and/or tumour tissue.

4.3 Secondary Objectives

To investigate preliminary efficacy of C225-ILs-dox in patients with relapsed EGFR amplified glioblastoma.

4.4 Safety Objectives

To investigate tolerability of C225-ILs-dox in patients with relapsed/refractory glioblastoma according to the CTCTAE criteria (18) version 4.0.

5. STUDY OUTCOMES

5.1 Primary Outcome

In this pharmacokinetic study, the main endpoint will be the ratio of the C225-ILs-dox concentration in the CSF over the C225-ILs-dox concentration in PB.

5.1.1 Measurement of pharmacokinetics

Peripheral blood

The methods of PK measurement have been standardized and described previously. To measure the concentration of doxorubicin and cetuximab-Fab′, we will first generate plasma from PB samples by centrifugation and separate one part of the plasma by size-exclusion chromatography on a cross-linked agarose gel filtration medium (Sepharose CL-4B column, GE Healthcare Bio-Sciences AB, Uppsala, Sweden) into fractions of greater than 20 megadaltons (liposomal fraction) and less than 20 megadaltons (free doxorubicin, free antibody, or antibody fragments). We will process the other part of the plasma without separation (total plasma). All samples will be analysed for doxorubicin and cetuximab-Fab′ concentrations. We will calculate doxorubicin concentrations with solid phase extraction followed by high-performance liquid chromatography (HPLC; LaChrom, Merck-Hitachi, Darmstadt, Germany). We will use a Luna 5μ Phenyl-Hexyl column (Phenomenex, Torrance, CA, USA) and measure doxorubicin peaks at 505/550 nm Ex/Em. Cetuximab-Fab′ will be detected and quantified by indirect ELISA (Spectra Max 190, Molecular Devices, Biberach, Germany). We will coat microtitre plates with the antigen EGFR and incubate the plates with plasma samples. We will quantify a secondary anti-human IgG antibody conjugated to peroxidase that binds to cetuximab-Fab′ by measuring the optical density of the substrate of tetramethylbenzidine.

CSF

We will use the same techniques as described above to assess the concentration of doxorubicin and cetuximab-Fab′, but the initial step of centrifugation will be omitted, and analyses will all be made on total CSF.

Tumour tissue

In those patients who underwent resection, the doxorubicin and doxorubicinol concentration in tumour tissue as well as in liquor will be determined by liquid chromatography (Shimadzu, Kyoto, Japan) tandem mass spectrometry (API 5500, AB Sciex, MA, USA). The method will be optimized in order to reach low drug concentrations (approximately 0.1-1 ng/mL). Moreover, the method accuracy and precision should be between 85-115% (Lower limit of quantification (LLOQ): 80-120%) and ≤15% (LLOQ: ≤20%), respectively.

5.2 Secondary Outcomes

- Tumour response according to RANO criteria (2) on the final MRI scan after completion of 4 treatment cycles (cycle 4, day 28).
- Best achieved tumour response (1st or second MRI scan) during treatment phase according to RANO criteria (2).
- Event free survival, defined as the time between registration to progression, termination of therapy for toxicity, or death whichever occurs first.
- Progression free survival, defined as the time between registration to progression or death whichever occurs first.
- Overall survival, defined as the time between registration to death due to any cause.
- Toxicity as graded by the CTCAE Version 4.0.

5.3 Other Outcomes of Interest

N/A

5.4 Safety Outcomes

The most frequent adverse events related to pegylated liposomal doxorubicin are:

Hand-foot syndrome: HFS, or palmar-plantar erythrodysesthesia, is characterized by tenderness and redness of the skin; in more severe cases small sores may appear. It usually occurs on the palms of the hands and soles of the feet, but may also occur on other parts of the body where friction, pressure, or warmth occur.

The occurrence is dependent on both the size of the single dose and on the dose schedule. HFS is the most frequently reported side effect with PLD treatment; in randomized studies its incidence ranges between 16 and 23% (grade 3-4).

Infusion-related reactions: Some patients may experience acute reactions when receiving the PLD infusion. It can appear as flushing, erythema, shortness of breath, back pain, tightening in the chest or throat. In randomized studies with PLD, infusion-related reactions were seen in 11% of the patients. The reactions are reversible, and if they appear, they are mainly seen in the first cycle of treatment and not thereafter.

Other toxicities: Several side effects that are associated with conventional doxorubicin are seen to a significantly lesser extent with PLD treatment. Cardiotoxicity is perhaps the most important of these. The cardiotoxic effects of conventional doxorubicin can result in irreversible damage, and its cumulative nature has led to recommendations not to exceed an accumulated life-time dose of 550 mg/m^2 of conventional doxorubicin. No such dose limit has been established for PLD. Other side effects seen less frequently with PLD are nausea, vomiting, alopecia and neutropenia. On the other hand, HFS, mucositis and stomatitis occur more frequently with PLD than with conventional doxorubicin.

For further details see the latest version of Swissmedic-approved product information of Caelyx™.

6. STUDY DESIGN

Prospective, single-stage, single-arm, open-label pharmacokinetic study at two Swiss centres

6.1 General study design and justification of design

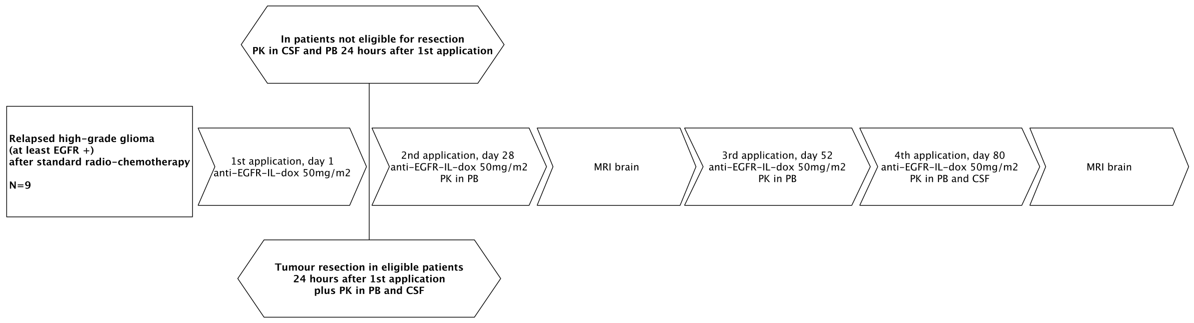

FIGURE *1*: Trial scheme for treatment and pharmacokinetic analyses. Abbreviations: CSF = cerebro-spinal fluid, PB = peripheral blood, PK = samples for pharmacokinetics.

There is no data on CSF penetration of C225-ILs-dox into the CSF compartment. There is also no evidence for efficacy of C225-ILs-dox in EGFR amplified glioblastoma.

To determine the pharmacokinetics regarding CSF penetration and the capacity of anti-EGFR-IL-dox to target the tumour tissue of patients with high-grade gliomas; nine patients will be included. All patients will be discussed at an interdisciplinary neuro-oncology tumour board at diagnosis of relapse. Irrespective of eligibility for resection of relapsed disease, patients will be evaluated for study eligibility. We aim to include at least 3 patients with resectable disease to assess anti-EGFR-IL-dox concentration in the tumour.

24 hours after the first intravenous application of anti-EGFR-IL-dox, *patients with resectable tumour* will undergo resection and have PK samples taken from PB and CSF (during operation). For those *patients with relapsed tumours, but deemed unresectable*, 24 hours after first application of anti-EGFR-IL-dox, PK samples from peripheral blood (venous puncture) and CSF (by lumbar puncture) will be taken.

All PK samples (PB, CSF and resected tumour tissue) will be assessed for the presence of anti-EGFR-IL-dox (see 5.1.1.).

There will be one MRI brain scan for interim assessment after the 2nd cycle (day 14). After the final MRI brain scan (cycle 4, day 28), further imaging is at the discretion of the treating physician. We will collect safety data until 3 months after last application.

Afterwards, patients enter the follow-up phase.

6.2 Methods of minimising bias

N/A

7. STUDY POPULATION

7.1 Eligibility criteria

7.1.1 Inclusion criteria

1. Written informed consent according to ICH/GCP regulations before registration and prior to any trial specific procedures
2. Patients with relapsed histologically proven glioblastoma ≥ 18 years of age.
3. Patients need to have at least one line of treatment with combined radio-chemotherapy
4. EGFR amplification. EGFR amplification will be tested by comparative genomic hybridization (CGH) method. <u>EGFR will be considered amplified if the value is 0.15 above the average signal of chromosome 7.</u>
5. Evaluable disease on MRI brain scan
6. Adequate bone marrow function: neutrophils ≥ 1.5 x 10^9/L, platelets ≥ 100 x 10^9/L
7. Adequate hepatic function: bilirubin ≤ 1.5 x ULN, AST, ALT and AP ≤ 2.5 x ULN
8. Adequate renal function: serum creatinine ≤ 1.5 x ULN and calculated creatinine clearance > 30 mL/min, according to the formula of Cockcroft-Gault, see Appendix 1.
9. Adequate cardiac function: Left ventricular Ejection Fraction (LVEF) ≥ 50% as determined by either echocardiography (ECHO) or radionuclide angiocardiography (MUGA) in addition to pre-BNP from peripheral blood
10. ECOG performance status of 0-2 (see Appendix 2)
11. No contraindications for lumbar puncture
12. Women with child-bearing potential have to use effective contraception, are not allowed to be pregnant and have to agree not to become pregnant during trial treatment and during the 6 months thereafter. A negative pregnancy test before inclusion into the trial is required for all women with child-bearing potential.

7.1.2 Exclusion criteria

1. History of hematologic or primary solid tumor malignancy, unless in remission for at least 3 years from registration except for adequately treated cervical carcinoma in situ and localized non-melanoma skin cancer.
2. Lack to provide written informed consent
3. Previous therapy with more than 240 mg/m^2 of doxorubicin or more than 450 mg/m^2 of epirubicin

4. Life expectancy less than 2 months
5. Any serious underlying medical condition (at the judgement of the investigator) which could impair the ability of the patient to participate in the trial (e.g. active autoimmune disease, uncontrolled diabetes, etc.)
6. Breastfeeding and pregnancy
7. Participation in any investigational drug trial within 4 weeks preceding treatment start
8. Any concomitant drugs contraindicated when administering Erbitux™ or Caelyx™ according to the Swissmedic-approved product information
9. Known hypersensitivity to trial drug(s) or to any component of the trial drug(s)
10. Any other serious underlying medical, psychiatric, psychological, familial or geographical condition, which in the judgment of the investigator may interfere with the planned staging, treatment and follow-up, affect patient compliance or place the patient at high risk from treatment-related complications.

7.2 Recruitment and screening

Patients will be screened and recruited by the treating oncologist. The patient will be presented at an interdisciplinary tumour board to assess potential resectability. There will be no financial compensation to study participants.

7.3 Assignment to study groups

N/A

7.4 Criteria for withdrawal / discontinuation of participants

Patients have the right to refuse further treatment for any reason and at any time. Patients who decide to withdraw from the trial will be informed that all data collected until the time point of their withdrawal will be used. For the patient's security, a last examination should be performed. Patients may be withdrawn at any time from trial treatment at the discretion of the investigator due to a SAE, or based on any other relevant medical condition. The patient will then be transferred to the follow-up phase.

In case patients drop out or withdraw before the pharmacokinetic sample has been taken, we recruit additional patients to reach the planned sample size.

8. STUDY INTERVENTION

8.1 Identity of Investigational Products

The C225-ILs-dox will be provided free of charge by the University Hospital Basel (Sponsor). The pharmacy of the University Hospital of Basel will manufacture and dispatch the C225-ILs-dox vials.

8.1.1 Experimental Intervention

C225-ILs-dox

8.1.2 Control Intervention

N/A

8.1.3 Packaging, Labelling and Supply (re-supply)

The sites will receive an initial stock at activation and then be responsible for reordering.

The C225-ILs-dox will be supplied for use as a solution of 0.5 mg doxorubicin HCl in 50 mL and 10 mL vials, for parenteral administration.

Storage area temperature conditions must be monitored and recorded daily. All temperature excursions must be immediately reported to the Sponsor.

C225-ILs-dox may be sensitive to shear-induced stress (e.g. agitation or rapid expulsion from the syringe). Vigorous handling (such as shaking) of C225-ILs-dox solution may results in aggregation of the protein and may create cloudy solutions. Vials are designated for single use only.

The C225-ILs-dox should be added to 500 mL of 5% glucose for injection. This formulation should be used within 24 hours after dilution in glucose. C225-ILs-dox dilution should be a clear and reddish solution without any signs of aggregation. For further details on C225-ILs-dox handling refer to its Investigator's Brochure.

The IMP will be labeled as presented below

Trial GBM-LIPO FOR CLINICAL TRIAL USE ONLY

Solution of anti-EGFR-ILs-dox for parenteral administration **10 ml** with doxorubicin-HCl 0,5 mg/ml

Store between 2-8 °C (DO NOT FREEZE), Protect from light

LOT: 000000S00 EXP: MM.JJJJ

Patient UPN: ____________

Investigator: ____________

Sponsor: University Hospital Basel – Division of Oncology,
Petersgraben 4, CH - 4031 Basel, Tel.: +41 61 265 50 74

Trial GBM-LIPO FOR CLINICAL TRIAL USE ONLY

Solution of anti-EGFR-ILs-dox for parenteral administration **50 ml** with doxorubicin-HCl 0,5 mg/ml

Store between 2-8 °C (DO NOT FREEZE), Protect from light

LOT: 000000S00 EXP: MM.JJJJ

Patient UPN: ____________

Investigator: ____________

Sponsor: University Hospital Basel – Division of Oncology,
Petersgraben 4, CH - 4031 Basel, Tel.: +41 61 265 50 74

8.2 Product quality and complaint handling

A PQC is defined as any suspicion of a product defect related to manufacturing, labelling, packaging or shipment (e.g. wrong storage condition during transport). A PQC may have an impact on the safety and efficacy of the product. Timely, accurate, and complete reporting and analysis of PQC information from clinical trials are crucial for the protection of patients, investigators and the sponsor, and are mandated by regulatory agencies worldwide.

In case site staff identifies a potential PQC situation, the product is placed in quarantine and Sponsor must be contacted immediately. In case a patient identifies a PQC situation, the investigator must be contacted immediately.

If the defect is combined with a SAE/SAR, the site staff must additionally report the event to the Sponsor according to the reporting timelines for SAEs. The Sponsor will decide how to proceed with the suspected product.

8.2.1 Storage Conditions

The C225-ILs-dox vials should be stored at a temperature ranging from 2 to 8°C to ensure optimal retention of physical and biochemical integrity. It is important not to freeze the trial drug, since liposomes will be disrupted.

8.3 Administration of experimental intervention

8.3.1 Schedule

The doxorubicin-loaded anti-EGFR-immunoliposomes (C225-ILs-dox) will be administered on day 1 of each 28-day cycle.

8.3.2 Dose calculation and administration

C225-ILs-dox are given intravenously at a dose of 50 mg/m^2 doxorubicin HCl over 60 min. The C225-ILs-dox should be added to 500 mL of 5% glucose for injection. The dosage is based on the MTD determined in the phase I first in man trial (16).

8.3.3 Premedication or supportive medication

Antiemetics and other medication considered necessary for the patient's safety and well-being may be given at the discretion of the investigator.

8.3.4 Warnings and precautions

The following precaution is valid for women taking part in this clinical trial with child-bearing potential: doxorubicin hydrochloride is suspected to cause serious birth defects when administered during pregnancy. Trial participants must use effective contraception during trial treatment and 6 months after the treatment stop. Please refer to Investigator's Brochure for further warnings and precautions.

Patients should be assessed clinically for toxicity prior to, during, and after each infusion. If unmanageable toxicity occurs, dose modifications and guidelines must be followed as provided below:

- Doses omitted for toxicity are not replaced
- Once the dose has been reduced for any type of toxicity, it should never be increased afterwards (except for reductions in case of hyperbilirubinemia).
- If necessary, infusions may be delayed for up to 4 weeks, this may especially occur in case of recovery from surgical resection. If *drug related AEs* are not resolved to grade 0 or 1 within that time, treatment must be discontinued. In case the patient has experienced and sustained AEs higher than grade 1, but clearly associated with the surgical intervention, the local investigator should discuss drug treatment continuation with the coordinating investigator.
- Refer to section 8.4 for AE-specific dose modifications.
- In case of conflicting recommendations, use the most restrictive treatment adjustment.
- The coordinating investigator should be contacted in case the investigator has any doubts about treatment delays and/or dose reductions.

8.3.5 Control Intervention

N/A

8.4 Dose modifications

The immediate management of any adverse event should be according to standard clinical practice for that event. Subsequent management of treatment-related adverse events should be guided by the investigator's assessment of causality

8.4.1 Hand-foot syndrome

Grade	Anti-EGFR-ILs
CTCAE grade 1<br>Minimal skin changes or dermatitis (e.g. erythema) without pain	No modification, unless the patient has experienced previous grade 3 or 4 HFS. If so, delay until resolved and continue with 75% of initial dose.
CTCAE grade 2<br>Skin changes (e.g. peeling, blisters, bleeding, oedema) or pain, not interfering with function	Delay until resolved to grade 0 or 1. Continue treatment at previous dose.<br>If patient has experienced previous grade 2 or 3 HFS, continue with 75% of initial dose.
CTCAE grade 3<br>Ulcerative dermatitis or skin changes with pain interfering with function	Delay until resolved to grade 0 or 1. Continue with 75% of initial dose.<br>Second occurrence: permanently discontinue treatment.
CTCAE grade 4	Permanently discontinue treatment.

<table>
<tr><th>Grade</th><th>Anti-EGFR-ILs</th></tr>
<tr><td colspan="2">In patients who develop HFS grade 1 or 2, topical steroid treatment (creams, ointments, etc.), vitamin B6, and local protective measures (gloves etc.) should be used.
For HFS grade 3, patients can be treated with prednisone (or equivalent) p.o. in addition to the above-mentioned local measures.</td></tr>
</table>

8.4.2 Mucositis/stomatitis

<table>
<tr><th>Grade</th><th>Anti-EGFR-ILs</th></tr>
<tr><td>CTCAE grade 1
Asymptomatic or mild symptoms; intervention not indicated</td><td>No modification, unless the patient has experienced previous grade 3 or 4 mucositis/stomatitis. If so, delay until resolved and continue with 75% of initial dose</td></tr>
<tr><td>CTCAE grade 2
Moderate pain; not interfering with oral intake; modified diet indicated</td><td>Delay until resolved to grade 0 or 1. Continue treatment at previous dose.
If patient had experienced previous grade 2-4 mucositis/stomatitis, continue with 75% of initial dose.</td></tr>
<tr><td rowspan="2">CTCAE grade 3
Severe pain; interfering with oral intake</td><td>Delay until resolved to grade 0 or 1. Continue with 75% of initial dose.</td></tr>
<tr><td>Second occurrence: permanently discontinue treatment.</td></tr>
<tr><td>CTCAE grade 4
Life-threatening consequences; urgent intervention indicated</td><td>Permanently discontinue treatment.</td></tr>
</table>

8.4.3 Haematological toxicity

<table>
<tr><th>Grade</th><th>Anti-EGFR-ILs</th></tr>
<tr><td>CTCAE grade 1 or 2</td><td>No action</td></tr>
<tr><td>CTCAE grade 3
Neutrophils 1.0 - 0.5 x 10^9/l or
Platelets 50 - 25 x 10^9/l</td><td>Delay until recovered to grade 0 or 1.
No dose reduction.</td></tr>
<tr><td rowspan="2">CTCAE grade 4
Neutrophils < 0.5 x 10^9/l or
Platelets < 25 x 10^9/l</td><td>Delay until recovered to grade 0 or 1. Continue with 75% of initial dose.</td></tr>
<tr><td>Second occurrence: permanently discontinue treatment.</td></tr>
<tr><td colspan="2">In case of anaemia, consider use of erythropoietin or erythrocytes concentrate infusions as clinically indicated and according to local guidelines.</td></tr>
</table>

8.4.4 Infection

<table>
<tr><th>Grade</th><th>Anti-EGFR-ILs</th></tr>
<tr><td>CTCAE grade 1, 2 or 3</td><td>No action</td></tr>
<tr><td rowspan="2">CTCAE grade 4
febrile neutropenia</td><td>Delay until recovered</td></tr>
<tr><td>Second occurrence: permanently discontinue treatment.</td></tr>
<tr><td colspan="2">In case of febrile neutropenia, consider the use of G-CSF according to local guidelines.</td></tr>
</table>

8.4.5 Cardiotoxicity (decrease of LVEF)

Grade	Anti-EGFR-ILs
LVEF decrease to < 50% by ≤ 10% percentage points from baseline	Administer treatment, but repeat LVEF measurement after 4 weeks.
LVEF decrease to < 50% by > 10% percentage points from baseline	Delay 2 scheduled infusions. Repeat LVEF measurement after 4 weeks.<br>If LVEF drop confirmed, permanently discontinue treatment (repeat cardiac assessment to be performed at 3 months after treatment discontinuation). Otherwise, resume treatment.

8.4.6 Bilirubin

Grade	Anti-EGFR-ILs
Bilirubin ≥ 2 x ULN	Continue at 50% of initial dose.<br>The dose may be re-escalated at the next infusion if no adverse drug reactions occur.
Bilirubin ≥ 3 x ULN	Discontinue permanently

8.4.7 Other adverse events

Grade	Anti-EGFR-ILs
CTCAE grade 1 or 2	No action
CTCAE grade 3 or 4	First occurrence: delay until recovered to grade 0 or 1.<br>Continue with 75% of initial dose.
	Second occurrence: permanently discontinue treatment

8.5 Compliance with study intervention

Compliance is not expected to be an issue. Patients in this situation are regularly seen by the treating oncologist for supportive care. The treatment planned in this study is given intravenously in the outpatient clinic so there will be no issue of drug accountability. In case patients do not attend the scheduled study visits, it is to the discretion of the treating oncologist to withdraw the patient from study.

8.6 Data Collection and Follow-up for withdrawn participants

Patients withdrawing from study will have a final safety visit. Data obtained during study until withdrawal will be used for analysis unless the patient clearly declines use of data collected in study. After withdrawal from study no further follow-up will be conducted.

8.7 Trial specific preventive measures

Treatments listed below are not permitted during the trial treatment phase. If they are medically required during treatment phase, the patient will be directly transferred to the follow-up phase.

- Other anticancer treatments
- Investigational treatments within other trials

8.8 Concomitant Interventions (treatments)

All drugs/interventions (e.g. pain medication, anti-epileptic medication, steroids, physiotherapy, anti-emetics, anti-infective drugs) required for supportive care are allowed.

8.9 Study Drug Accountability

Sites must report the reception, dispensing and destruction for C225-ILs-dox.

If sites already have their own accounting system, it may be used instead of the Sponsor's drug inventory log only after inspection and approval by the CRA.

The investigator must maintain 100% accountability for all trial medication received and dispensed during the entire participation in the trial. Proper drug accountability includes:

- Continuously monitoring of expiry dates
- Verifying that the drug inventory log and the drug dispensing log are completed accurately and legibly

If any dispensing errors or discrepancies are discovered, Sponsor must be notified immediately.

8.10 Return or Destruction of Study Drug

Unused, partly unused or expired trial medication must be destroyed at the site according to local guidelines, but only after Sponsor's approval.

9. STUDY ASSESSMENTS

To determine the pharmacokinetics regarding CSF penetration and the capacity of anti-EGFR-IL-dox to target the tumour tissue of patients with high-grade gliomas; nine patients will be included. All patients will be discussed at an interdisciplinary neuro-oncology tumour board at diagnosis of relapse. Irrespective of eligibility for resection of relapsed disease, patients will be evaluated for study eligibility. We aim to include at least 3 patients with resectable disease to assess anti-EGFR-IL-dox concentration in the tumour.

24 hours after the first intravenous application of anti-EGFR-IL-dox, *patients with resectable tumour* will undergo resection and have PK samples taken from PB and CSF (during operation). For those *patients with relapsed tumours, but deemed unresectable*, 24 hours after first application of anti-EGFR-IL-dox, PK samples from peripheral blood (venous puncture) and CSF (by lumbar puncture) will be taken.

All PK samples (PB, CSF and resected tumour tissue) will be assessed for the presence of anti-EGFR-IL-dox.

There will be one MRI brain scan for interim assessment after the 2nd cycle (day 14). After the final MRI brain scan (cycle 4, day 28), further imaging is at the discretion of the treating physician. We will collect safety data until 3 months after last application.

Afterwards, patients enter the follow-up phase.

9.1 Study flow chart

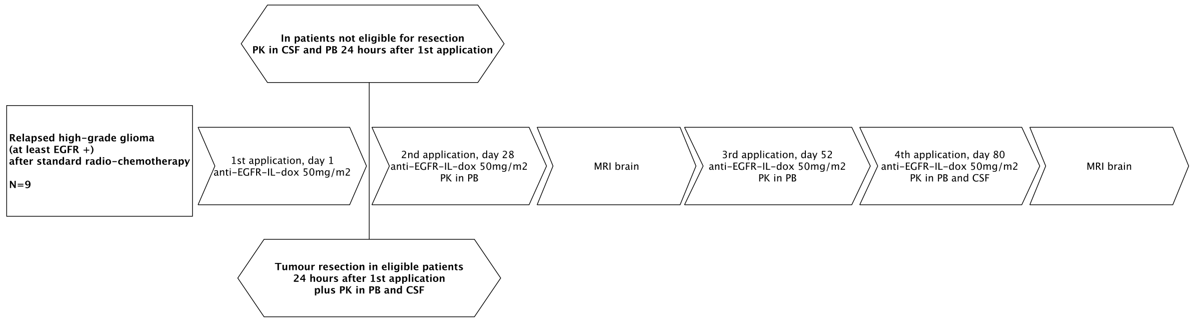

FIGURE *2*: Trial scheme for treatment and pharmacokinetic analyses. Abbreviations: CSF = cerebro-spinal fluid, PB = peripheral blood, PK = samples for pharmacokinetics.

Planned duration of treatment is 4 months (Day 1 of cycle 1 until day 28 of cycle 4; cycles given in 28 day intervals). The treatment phase finishes with the final MRI scan on day 28 of the 4th cycle. Afterwards, patients are transferred into the follow-up phase.

In case one of the following events occurs during the treatment phase, patients are directly transferred into the follow-up phase:

- progressive disease
- unacceptable toxicity
- patient refusal
- withdrawal by the physician
- protocol treatment was delayed for more than 4 weeks (also for vacation)
- start of a not permitted treatment

- patient becomes pregnant

10. REGISTRATION

- Check the central EGFR assessment, only patients with EGFR amplified tumours will be registered into the trial
- Obtain written informed consent from the patient for participation in the trial

Patients can be registered directly via Internet (accessible 24 hours a day, 7 days a week) using the following web address:

https://secutrial2.usb.ch/apps/WebObjects/ST21-productive-DataCapture.woa/wa/choose?customer=ONK

After patient registration

- Report the baseline clinical and laboratory information, baseline symptoms and baseline tumor assessment in the CRFs
- Update the screening and enrollment list and fill in the patient identification list
- The signed Eligibility Form (printout of CRF subform ER) has to be sent to the Sponsor by mail or fax within one month after registration. The original is kept in the investigator's file
- Trial therapy should be started within 7 days from registration.

11. ASSESSMENTS OF OUTCOMES

11.1 Assessment of primary outcome

We will assess anti-EGFR-IL-dox concentration in the CSF 24 hours after first intravenous application of anti-EGFR-IL-dox by lumbar puncture or in the tumour tissue (only in three patients) and peripheral blood. In parallel, we will measure anti-EGFR-IL-dox in the peripheral blood taken together with routine laboratory tests at each of the remaining three treatment cycles.

11.2 Assessment of secondary outcomes

We will conduct two MRI brain scans to assess anti-EGFR-IL-dox efficacy.

11.3 Assessment of other outcomes of interest

N/A

11.4 Assessment of safety outcomes

Safety outcomes will be monitored throughout the study (treatment and follow-up phase).

11.4.1 Adverse events

Regarding adverse events, we will collect: time of onset, duration, resolution, action been taken, assessment of intensity, relationship with study treatment (see section 10) at each treatment visit every 28 days and at any time at acute onset.

11.4.2 Laboratory parameters

At each treatment visit, we will collect laboratory parameters capturing liver function and kidney function as well as . We will furthermore assess possible haematological toxicity by collecting full blood counts. All these measures will be taken and analysed at the respective study site.

11.4.3 Vital signs

Vital signs such as body temperature, blood pressure and heart beat will be assessed at each treatment visit (every 28 days).

11.4.4 End of treatment visit

The end of treatment visit takes place after application of the last cycle (Cycle 4, day 28). As safety parameter, we will take a full blood count and pro-BNP.

11.4.5 Evaluation in follow-up phase

After the end of treatment visit, patients are followed-up every 6 weeks with haematology and pro-BNP. Other investigations (including imaging) will be conducted as clinically indicated.

11.5 Assessments in participants who prematurely stop the study

Describe follow-up procedures and assessments in participants who are withdrawn from the study prematurely (e.g., recording of adverse events, physical examination, laboratory parameters, vital signs). Define follow-up period; refer to Section 10 for procedures for participants who prematurely stop the study.

11.6 Procedures at each visit

11.6.1 To be performed before registration (Screening):

EGFR amplification on the primary tumour sample will be performed by comparative genomic hybridization or by fluorescence in situ hybridization (FISH) by the neuropathology at the University Hospital in Basel and in collaboration with the neuropathology in Bonn.

11.6.2 Can be performed within 28 days before registration (Screening, day -28 to -1):

- Medical history including baseline symptoms, previous therapies, location of tumour sites in the brain
- Urine pregnancy test for women with child-bearing potential
- Tumour assessments by MRI imaging of the brain
- Echocardiography, pro-BNP and ECG

11.6.3 To be performed within 7 days before registration (Screening, day -7 to -1):

- Vital signs and physical measurements (blood pressure, heart rate, height, weight, ECOG performance status and physical examination)
- Haematology: haemoglobin, neutrophils, platelets
- Biochemistry: calcium, potassium, sodium, magnesium
- Hepatic function: AP, AST, ALT, bilirubin
- Renal function: serum creatinine, calculated creatinine clearance (according to the formula of Cockcroft-Gault, see Appendix 1)

11.6.4 To be performed at each treatment visit (every 4 weeks, treatment phase ± 5 days)

In case investigations of 11.6.3 have been conducted within 3 days before the first treatment application, the investigations mentioned below do not have to be repeated.

- Vital signs and physical measurements (blood pressure, heart rate, ECOG performance status and physical examination, body temperature)
- Haematology: haemoglobin, neutrophils, platelets
- Biochemistry: calcium, potassium, sodium, magnesium
- Hepatic function: AP, AST, ALT, bilirubin
- Renal function: serum creatinine, calculated creatinine clearance (according to the formula of Cockcroft-Gault, see Appendix 1)
- Peripheral blood sample for PK analysis
- Clotting tests - only on the day of first treatment application before the lumbar puncture (according to local standards) for collection of CSF

11.6.5 Lumbar puncture after 1st treatment application

- Only after the 1st treatment application
- 4 ml CSF by lumbar puncture for PK analysis 24 hours after first application

11.6.6 Surgical resection of relapsed tumour

Only applies to patients in which the relapsed tumour is deemed resectable by the neurosurgical team

- Resection to be performed around 24 hours after 1st treatment application

11.6.7 To be performed at the end of treatment visit (28 days after last treatment application ± 5 days)

At the end or treatment visit we will investigate the following parameters:

- Vital signs and physical measurements (ECOG performance status and physical examination)
- Haematology: haemoglobin, neutrophils, platelets
- Biochemistry: calcium, potassium, sodium, magnesium
- Cardiac function: proBNP
- Hepatic function: AP, AST, ALT, bilirubin
- Renal function: serum creatinine, calculated creatinine clearance (according to the formula of Cockcroft-Gault, see Appendix 1)

11.6.8 To be performed at each visit during follow-up phase (every 6 weeks ± 5 days for 12 months)

After completion of the treatment-phase, patients come into follow-up phase which lasts 12 months at maximum. It starts 28 days after day 1 of cycle 4. During follow-up phase, patients have regular follow-up visits every 6 weeks.

- Vital signs and physical measurements (ECOG performance status and physical examination)
- Haematology: haemoglobin, neutrophils, platelets
- Biochemistry: calcium, potassium, sodium, magnesium
- Hepatic function: AP, AST, ALT, bilirubin
- Renal function: serum creatinine, calculated creatinine clearance (according to the formula of Cockcroft-Gault, see Appendix 2)

11.6.9 To be performed at the end of treatment visit (8 weeks after last follow-up visit)

- Vital signs and physical measurements (ECOG performance status and physical examination)
- Cardiac function: proBNP

12. SAFETY

12.1 Drug studies

During the entire duration of the study, all adverse events (AE) and all serious adverse events (SAEs) are collected, fully investigated and documented in source documents and case report forms (CRF). Study duration encompassed the time from when the participant signs the informed consent until the last protocol-specific procedure has been completed, including a safety follow-up period.

12.1.1 Definition and assessment of (serious) adverse events and other safety related events

12.1.1.1 Adverse Event

An **Adverse Event (AE)** is any untoward medical occurrence in a patient or a clinical investigation participant administered a pharmaceutical product and which does not necessarily have a causal relationship with the study procedure. An AE can therefore be any unfavourable and unintended sign (including an abnormal laboratory finding), symptom, or disease temporally associated with the use of a medicinal (investigational) product, whether or not related to the medicinal (ivestigational) product.

12.1.1.2 Reporting of AEs

Patients will be instructed by the investigator to report the occurrence of any AE. The investigator assesses and records all AEs observed during the AE reporting period from registration until 30 days after end of treatment. Ongoing AEs need to be followed up until resolution or stabilization with the expectation that it will remain chronic or start of new line of therapy.

12.1.1.3 Adverse events observed with C225-ILs-dox

Most adverse events and SAEs in the phase I trial (16) of doxorubicin loaded anti-EGFR immunoliposomes were due to pre-existing conditions or tumour progression with worsening of tumour-

related symptoms such as bleeding from the tumour site, renal failure due to compression of both ureters, pre-existing pain, and gastrointestinal perforation in the presence of clearly progressive gastric cancer. Three SAEs resulted in death. Only three SAEs (febrile neutropenia, septicaemia, and a fatal massive oral bleed) were probably or possibly related to the trial drug.

12.1.1.4 Serious Adverse Event

A **Serious Adverse Event (SAE)** is classified as any untoward medical occurrence that:

- results in death,
- is life-threatening,
- requires in-patient hospitalization or prolongation of existing hospitalisation,
- results in persistent or significant disability/incapacity, or
- is a congenital anomaly/birth defect.
- A hospitalization (>24 hours) for the following circumstances is not to be reported as a SAE:
    - elective surgery (planned before entry into the trial)
    - protocol related treatments, procedures or monitoring
    - social reasons (e.g. in rehabilitation home)
    - progressive disease

In addition, important medical events that may not be immediately life-threatening or result in death, or require hospitalisation, but may jeopardise the patient or may require intervention to prevent one of the other outcomes listed above will also be considered serious.

Examples of such events are intensive treatment in an emergency room or at home for allergic bronchospasm, blood dyscrasias or convulsions that do not result in hospitalisation, or development of drug dependency or drug abuse.

SAEs should be followed until resolution or stabilisation. Participants with ongoing SAEs at study termination (including safety visit) will be further followed up until recovery or until stabilisation of the disease after termination.

12.1.1.5 Assessment of Causality

Both Investigator and Sponsor-investigator make a causality assessment of the event to the study drug, based on the criteria listed in the ICH E2A guidelines:

Relationship	Description
Definitely	Temporal relationship<br>Improvement after dechallenge*<br>Recurrence after rechallenge<br>(or other proof of drug cause)
Probably	Temporal relationship<br>Improvement after dechallenge<br>No other cause evident
Possibly	Temporal relationship<br>Other cause possible
Unlikely	Any assessable reaction that does not fulfil the above conditions
Not related	Causal relationship can be ruled out
*Improvement after dechallenge only taken into consideration, if applicable to reaction	

12.1.1.6 Unexpected Adverse Drug Reaction

An “unexpected” adverse drug reaction is an adverse reaction, the nature or severity of which is not consistent with the applicable product information (e.g. Investigator’s Brochure for drugs that are not yet approved and Product Information for approved drugs, respectively). [ICH E2A]

12.1.1.7 *Suspected Unexpected Serious Adverse Reactions (SUSARs)*

The Sponsor-Investigator evaluates any SAE that has been reported regarding seriousness, causality and expectedness. If the event is related to the investigational product and is both serious and unexpected, it is classified as a SUSAR.

12.1.1.8 *Assessment of Severity*

AEs are coded with the NCI Common Terminology Criteria for Adverse Events (CTCAE) v4.0, and assigned a grade (from 1 = mild to 5 = death related to AE) as well as a relationship to trial treatment. The NCI CTCAE v4.0 (as pdf) as well as instructions on how to use the criteria can be found on http://ctep.cancer.gov/protocolDevelopment/electronic_applications/ctc.htm.

Note:

- Report the start and end date of the event and any changes in grading observed within the reporting period.
- Baseline symptoms will be recorded on the CRF and will continue to be followed up during treatment.
- AEs are documented by the codes according to CTCAE v4.0. If none of the codes are applicable, it exists for each of the 26 SOCs the term 'others' to describe the AE. If the term 'others' is applicable, briefly describe the AE in a comprehensive and understandable manner.
- Laboratory values will be documented as absolute values on the CRFs. Out of range laboratory values occurring outside of predefined assessment times or any laboratory values not specifically asked to be assessed by the protocol should only be documented as AE if they are grade 3 or higher.

12.1.2 Reporting of serious adverse events (SAE) and other safety related events

Any SAE must be reported by submitting the completed **initial report** section of the trial-specific SAE form within 24 hours of becoming aware of the event. This form is available through SecuTrial.

Submission is done by sending the SAE form by fax to:

The SAE outcome must be reported within 2 weeks after initial report by submitting the **follow-up** report (e.g. initial SAE form, updated with follow-up information) to the Sponsor as above. In case the SAE is reported as ongoing after 14 days, the follow-up report must be submitted again with the final outcome.

The local investigator is responsible to inform his/her local EC about local SAEs that are fatal or resulted in death within 7 days.

12.1.3 Reporting of SAEs and SUSARs by the Sponsor

The Sponsor ensures that all reporting requirements and timelines for reporting, as defined in the applicable national law are followed.

The Sponsor will forward any SAE which is fatal and occurred at a Swiss site to the lead EC according to the Swiss Human Research Act (HRA) and its applicable Ordinances.

The Sponsor will report every SUSAR to all principal investigators, to the involved ECs, to Swissmedic as specified in the Swiss HRA.

The Sponsor will forward each individual SAE to the coordinating investigator.

12.1.4 Periodic reporting on safety to principal investigators

The Sponsor ensures that the reporting requirements and timelines for reporting, as defined in the respective applicable laws, are followed.

An annual safety report (ASR) will be provided to the local investigators for filing into the investigator's file. The Sponsor will submit the ASR to the involved ECs and to Swissmedic.

12.1.5 Reporting and Handling of Pregnancies

If applicable, describe the handling and reporting duties in case of a pregnancy during the study

Pregnant participants must immediately be withdrawn from the clinical study. Any pregnancy during the treatment phase of the study and within 30 days after discontinuation of study medication will be reported to the Sponsor-Investigator within 24 hours. The course and outcome of the pregnancy should be followed up carefully, and any abnormal outcome regarding the mother or the child should be documented and reported.

12.1.6 Follow up of (Serious) Adverse Events

In case patients experience an SAE and are withdrawn from the study, the SAE will be followed-up until resolved and reported as if the patient was on study. Only in case the patient declines further reporting to the Sponsor the follow-up of SAE will not be reported. However, the treating oncologist will be encouraged to tightly follow-up the patient as per local practise and document the progress in the local health record system.

12.1.7 Pregnancy

In the case of pregnancy occurring during trial treatment or within 6 months after end of trial treatment, the investigator must report the event to the Sponsor by completing the pregnancy reporting form. This form (available on SecuTrial) has to be filled in and faxed to the Sponsor within 24 hours. The investigator shall ensure that the case is followed up to the end of the pregnancy and supply a final report on the outcome to the Sponsor. In addition, the investigator has to report any foetal anomaly, stillbirth or any other medicinal significant event concerning the pregnancy as SAE.

13. STATISTICAL METHODS

13.1 Hypothesis

This is an exploratory study with no specifically defined hypothesis.

13.2 Determination of Sample Size

No sample size calculation was done for this exploratory pharmacokinetic study, since this a pharmacokinetic phase 1 study to demonstrate CNS availability of C225-ILs-dox. In order to faithfully predict the bioavilability of C225-ILs-dox and the concentration of active compounds in the CNS, 9 patients will be treated. We aim to collect of 3 patients resection material (patients undergoing surgery at the time point of relapse).Statistical criteria of termination of trial

No formal stopping rules based on statistical considerations have been established for this exploratory study.

13.3 Planned Analyses

13.3.1 Datasets to be analysed, analysis populations

We will describe the patient population baseline characteristics including previous treatments and time in remission after last treatment using descriptive statistics with frequencies (proportions) and means/medians (range) as appropriate.

13.3.2 Primary Analysis

Regarding the main endpoint (pharmacokinetics), at each time point (24 hours after first application and day 80) we will calculate:

- the ratio of the CSF concentration of doxorubicin (µg/ml) over the total plasma concentration of doxorubicin (µg/ml) [$CSF_{dox}/Plasma_{dox}$]
- the ratio of the CSF concentration of the liposomal fraction (µg/ml) over the total plasma concentration of the liposomal fraction (µg/ml) [$CSF_{lipo}/Plasma_{lipo}$]
- the ratio of the CSF concentration of the cetuximab-Fab′ (ng/ml) over the total plasma concentration of the cetuximab-Fab′ (ng/ml) [$CSF_{FAB}/Plasma_{FAB}$]

We will illustrate the ratios of concentration using plots showing the median/mean and range at each time point. Plots for CSF and peripheral blood concentrations will also be created separately. We will calculate the number (proportion) of patients in which no C225-ILs-dox concentration was detected in the CSF.

13.3.3 Secondary Analyses

Regarding tumour response, we will calculate frequency (proportions with all patients registered in the

denominator) of tumour response status as per RANO criteria: responders (complete response or partial response), non-responder (stable disease, progressive disease). Patients without any response assessment due to any cause will be considered as non-responders. Regarding time-to-event data, we will use the Kaplan-Meier product estimate to illustrate survival probabilities for EFS and OS. Toxicity events per patient will be summarized using descriptive statistics (frequencies, proportions).

Describe the intended subgroup analyses, if applicable, that will be done, when and how and by whom they will be done, add hypothesis related to each subgroup.

13.3.4 Interim analyses

No interim analysis planned for this study.

13.3.5 Safety analysis

We will summarize frequency (proportion), type and severity of reported AEs and SAEs as graded by the CTCAE version 4.0 in total and per patient.

13.3.6 Deviation(s) from the original statistical plan

In case of deviations from the statistical analysis plan, we will report these in any resulting publication. The protocol will be published in conjunction with any journal publication.

13.4 Handling of missing data and drop-outs

In case a patient drops out before the pharmacokinetic analysis, we will replace the patient. We will transparently such cases in an adequate patient flow diagram in any resulting publication.

14. QUALITY ASSURANCE AND CONTROL

Several procedures ensure the quality of the trial in compliance with applicable regulatory requirements, GCP and the protocol:

- Written standard operating procedures are implemented
- Personnel involved in conducting the trial is qualified by education, training and experience
- An updated staff list must be kept at the site (template available on the SAKK website)
- Validation of database and statistical analysis
- Quality control principles are implemented
- On-site and central monitoring to evaluate protocol compliance (SDV, verification of informed consent etc.) by personnel designated by the SAKK
- Data captured online will be validated in real-time, yielding errors (for inacceptable data) and warnings (for possibly inconsistent data - these warnings may be overruled by the user).
- Audit trail of changes
- Medical data review by the coordinating investigator or a delegated person (all CRFs will be reviewed and checked on medical content)
- Pathology review and central EGFR assessment
- An independent radiologic review of the tumor assessments (from baseline and at 12 months) only for the progression-free patients at 12 months if the trial results are positive
- Safety monitoring
- Accountability of the doxorubicin-loaded anti-EGFR-immunoliposomes
- Internal audit procedures
- Central management of deviations and implementation of corrective and preventive measures.

14.1 Data handling and record keeping / archiving

14.1.1 Investigator’s file

All trial-related correspondence should be filed in the investigator’s file.

14.1.2 Record retention

The site will retain all essential documents according to ICH GCP. This includes copies of the patient trial records, which are considered as source data, CRFs, patient informed consent statement,

laboratory printouts, drug inventory and destruction logs, and all other information collected during the trial. These documents will be stored for at least 10 years after the termination of the trial. The end of this retention period will be communicated to the sites by the Sponsor.

The site will retain all essential documents according to ICH GCP. This includes copies of the patient trial records, which are considered as source data, patient informed consent statement, laboratory printouts, drug inventory and destruction logs, and all other information collected during the trial. These documents were stored until at least 10 years after the termination of the trial. The end of this retention period will be communicated to the sites by the Sponsor**.** For the patient trial records, which are entered into the EDC system, the sponsor guarantees the access and availability of the data at any time at least 10 years after the termination of the trial. In the event that the principal investigator retires or changes employment, custody of the records may be transferred to another competent person who will accept responsibility for those records. Written notice of such transfer will be given to the Sponsor. The Sponsor will notify the concerned regulatory authorities.

14.1.3 Case Report Forms

14.1.3.1 *CRFs and reports*

CRFs specifically created for this trial are used for documentation. It is very important to adhere to the schedule of visits prescribed in the protocol for all patients. All CRFs needed for the corresponding visit will be displayed automatically in the SecuTrial system. The CRFs must be completed online in a timely manner. In general, the data should be entered into the CRFs within a month from the visit or medical examination. Sites must use a patient identification list to allow identification of a patient. This list must be kept at the site in the investigator's file.

14.1.3.2 *Eligibility CRF:*

- The completed sub form ER in the web based EDC system must be printed and signed by the investigator. **A copy of the signed sub form must be sent to the Sponsor by mail or fax within one month after registration.** The original signed form is kept at the site in the Investigator's file.
- If it is not possible to enter the eligibility form online, complete the paper CRF version and fax it to the Sponsor for registration of the patient (see also Section 7).

14.1.3.3 *SAE and pregnancy report forms:*

- Trial-specific SAE report forms and trial-specific pregnancy report forms must be submitted **via the SecuTrial** system to the Sponsor within 24 hours of becoming aware of the SAE or pregnancy. Originals of SAE and pregnancy reports are kept at the site in the Investigator's file.

14.1.4 Specification of source documents

Source data are all information in original records and certified copies of original records of clinical findings, observations, or other activities in a clinical trial necessary for the reconstruction and evaluation of the trial.

Additionally, the following data entered directly onto trial documents are considered to be source data:

- patient screening and enrollment list
- patient identification list
- drug inventory logs
- Histopathology report which must include a statement on high-grade characteristics of the glioma

14.1.5 Record keeping / archiving

For the patient trial records, which are entered into the SecuTrial system, the sponsor guarantees the access and availability of the data at any time at least 10 years after the termination of the trial. In the event that the principal investigator retires or changes employment, custody of the records may be transferred to another competent person who will accept responsibility for those records. Written notice of such transfer will be given to the Sponsor. The Sponsor will notify the concerned regulatory authorities.

14.2 Data management

eCRF data will be entered using the Electronic Data Capture software secuTrial® and stored on a dedicated Oracle® database server. The overall size of the data will not exceed 20~GB.

14.2.1 Data Management System

The data is managed in the secuTrial system.

14.2.2 Data security, access and back-up

The EDC system is accessible via a standard browser on devices with internet connection. The data transfer between clients and servers is encrypted using Transport Layer Encryption (TLS) cryptography protocol. Password protection and user-right management ensure that only authorized study investigators, monitors, data managers and local authorities (if necessary) will have access to the data during and after the study. User administration and user training is performed by the CTU Basel according to predefined processes. Built-in audit trail will register any unplanned deviations from the study protocol. Study data are only accessible by authorized persons.

14.2.3 Analysis and archiving

Once all data are entered into the EDC system and monitoring is completed, the secuTrial® database will be locked and closed for further data entry. The complete dataset is then exported and transferred to the study statistician as well as the principal investigator through a secure channel. The exported data will be archived for 10 years by the principal investigator.

14.2.4 Electronic and central data validation

The data managers of the CTU Basel will implement validation rules in the EDC system. When data gets saved in an eCRF, it will be validated for completeness and discrepancies. The data will be reviewed by the responsible investigator as well as an independent monitor. The monitor will raise queries using the query management system implemented in secuTrial®. Designated investigators have to respond to the query and confirm or correct the corresponding data. Thereafter the monitor can close the query.

14.3 Monitoring

See separate Monitoring plan

14.4 Audits and Inspections

Authorities have the right to perform inspections, and the Sponsor has the right to perform on-site auditing during working hours upon reasonable prior notice.

14.5 Confidentiality, Data Protection

Trial-related data of the patient will be provided in a coded manner to the Sponsor. A unique patient number (UPN) will be attributed to each patient registered into the trial.

Identification of patients must be guaranteed at the site. For this purpose, sites are requested to use the patient screening and enrollment and the patient identification lists specifically produced for the trial. To avoid identification errors, the year of birth and the UPN must be provided on the CRFs. Patient confidentiality will be maintained according to applicable legislation. Patients must be informed of, and agree to, data and material transfer and handling, in accordance with Swiss data protection law. All information concerning the IMPs in connection with this trial and not previously published is considered confidential and proprietary information.

14.6 Storage of biological material and related health data

No additional biological material will be saved in the study described herein. All samples taken for measuring PK will be destroyed after successful measurement.

15. PUBLICATION AND DISSEMINATION POLICY

Results of this study will be published irrespective of the nature of findings. Members of the protocol committee are primarily responsible to analyse the data, draft the manuscript and submit the work for publication to a peer-reviewed medical journal.

16. FUNDING AND SUPPORT .

16.1 Funding

This study is supported by a grant from the Swiss Cancer League (KFS-4129-02-2017).

16.2 Other Support

The IMP (C225-ILs-dox) is currently also tested in a multicentre phase II trial conducted by the SAKK and can be used for this study free of charge.

17. INSURANCE

Insurance will be provided by the Sponsor-Investigator (University Hospital Basel). A copy of the certificate is filed in each investigator site file and the trial master file.

18. REFERENCES

1. Wicki A, Ritschard R, Loesch U, Deuster S, Rochlitz C, Mamot C. Large-scale manufacturing of GMP-compliant anti-EGFR targeted nanocarriers: production of doxorubicin-loaded anti-EGFR-immunoliposomes for a first-in-man clinical trial. Int J Pharm. 2015 Apr;484(1–2):8–15.

2. Wen PY, Macdonald DR, Reardon DA, Cloughesy TF, Sorensen AG, Galanis E, et al. Updated response assessment criteria for high-grade gliomas: response assessment in neuro-oncology working group. J Clin Oncol Off J Am Soc Clin Oncol. 2010 Apr 10;28(11):1963–72.

3. Wen PY, Kesari S. Malignant gliomas in adults. N Engl J Med. 2008 Jul;359(5):492–507.

4. Ferlay J, Parkin DM, Steliarova-Foucher E. Estimates of cancer incidence and mortality in Europe in 2008. Eur J Cancer. 2010;46(4):765–781.

5. Stupp R, Mason WP, van den Bent MJ, Weller M, Fisher B, Taphoorn MJB, et al. Radiotherapy plus concomitant and adjuvant temozolomide for glioblastoma. N Engl J Med. 2005 März;352(10):987–96.

6. Stupp R, Tonn J-C, Brada M, Pentheroudakis G. High-grade malignant glioma: ESMO Clinical Practice Guidelines for diagnosis, treatment and follow-up. Ann Oncol Off J Eur Soc Med Oncol ESMO. 2010 Mai;21 Suppl 5:v190–3.

7. Stupp R, Taillibert S, Kanner AA, Kesari S, Steinberg DM, Toms SA, et al. Maintenance Therapy With Tumor-Treating Fields Plus Temozolomide vs Temozolomide Alone for Glioblastoma: A Randomized Clinical Trial. JAMA. 2015 Dezember;314(23):2535–2543.

8. Stupp R, Brada M, van den Bent MJ, Tonn J-C, Pentheroudakis G. High-grade glioma: ESMO Clinical Practice Guidelines for diagnosis, treatment and follow-up. Ann Oncol Off J Eur Soc Med Oncol ESMO [Internet]. 2014 Apr; Available from: http://www.ncbi.nlm.nih.gov/pubmed/24782454

9. Taal W, Oosterkamp HM, Walenkamp AME, Dubbink HJ, Beerepoot LV, Hanse MCJ, et al. Single-agent bevacizumab or lomustine versus a combination of bevacizumab plus lomustine in patients with recurrent glioblastoma (BELOB trial): a randomised controlled phase 2 trial. Lancet Oncol. 2014 Aug;15(9):943–53.

10. Friedman HS, Prados MD, Wen PY, Mikkelsen T, Schiff D, Abrey LE, et al. Bevacizumab alone and in combination with irinotecan in recurrent glioblastoma. J Clin Oncol Off J Am Soc Clin Oncol. 2009 Oktober;27(28):4733–40.

11. Kreisl TN, Kim L, Moore K, Duic P, Royce C, Stroud I, et al. Phase II trial of single-agent bevacizumab followed by bevacizumab plus irinotecan at tumor progression in recurrent glioblastoma. J Clin Oncol Off J Am Soc Clin Oncol. 2009 Feb;27(5):740–5.

12. Hatanpaa KJ, Burma S, Zhao D, Habib AA. Epidermal growth factor receptor in glioma: signal transduction, neuropathology, imaging, and radioresistance. Neoplasia N Y N. 2010 Sep;12(9):675–84.

13. Heimberger AB, Hlatky R, Suki D, Yang D, Weinberg J, Gilbert M, et al. Prognostic effect of epidermal growth factor receptor and EGFRvIII in glioblastoma multiforme patients. Clin Cancer Res Off J Am Assoc Cancer Res. 2005 Feb;11(4):1462–6.

14. Roth P, Weller M. Challenges to targeting epidermal growth factor receptor in glioblastoma: escape mechanisms and combinatorial treatment strategies. Neuro-Oncol. 2014 Oktober;16 Suppl 8:viii14–9.

15. Reardon DA, Lassman AB, van den Bent M, Kumthekar P, Merrell R, Scott AM, et al. Efficacy and safety results of ABT-414 in combination with radiation and temozolomide in newly diagnosed glioblastoma. Neuro-Oncol. 2017 Jul;19(7):965–975.

16. Mamot C, Ritschard R, Wicki A, Stehle G, Dieterle T, Bubendorf L, et al. Tolerability, safety, pharmacokinetics, and efficacy of doxorubicin-loaded anti-EGFR immunoliposomes in advanced solid tumours: a phase 1 dose-escalation study. Lancet Oncol. 2012 Dec;13(12):1234–41.

17. Mamot C, Drummond DC, Greiser U, Hong K, Kirpotin DB, Marks JD, et al. Epidermal growth factor receptor (EGFR)-targeted immunoliposomes mediate specific and efficient drug delivery to EGFR- and EGFRvIII-overexpressing tumor cells. Cancer Res. 2003 Jun 15;63(12):3154–61.

18. Common Terminology Criteria for Adverse Advents (CTCAE) version 4.0 [Internet]. Available from: https://evs.nci.nih.gov/ftp1/CTCAE/CTCAE_4.03_2010-06-14_QuickReference_5x7.pdf

19. APPENDICES

Appendix 1 Calculation of eGFR

The CKD-EPI equation performed better than the MDRD (Modification of Diet in Renal Disease Study) equation, especially at higher GFR, with less bias and greater accuracy. When looking at NHANES (National Health and Nutrition Examination Survey) data, the median estimated GFR was 94.5 mL/min per 1.73 m^2 vs. 85.0 mL/min per 1.73 m^2, and the prevalence of chronic kidney disease was 11.5% versus 13.1%.

The CKD-EPI equation, expressed as a single equation, is:

$$GFR = 141 * \min(Scr/\kappa,1)^{\alpha} * \max(Scr/\kappa, 1)^{-1.209} * 0.993^{Age} * 1.018 \text{ [if female]} * 1.159 \text{ [if black]}$$

Scr is serum creatinine (mg/dL), κ is 0.7 for females and 0.9 for males, α is -0.329 for females and -0.411 for males, min indicates the minimum of Scr/κ or 1, and max indicates the maximum of Scr/κ or 1.

Reference
The CKD-EPI was derived and validated by Levey et al. A New Equation to Estimate Glomerular Filtration Rate. Ann Intern Med. 2009;150:604-612.

Appendix 2 WHO performance status

Performance status should be calculated according to the ECOG/WHO definition (23).

0 Fully active, able to carry on all pre-disease performance without restriction.
1 Restricted in physically strenuous activity but ambulatory and able to carry out work of a light or sedentary nature, e.g., light house work, office work.
2 Ambulatory and capable of all self-care but unable to carry out any work activities. Up and about more than 50% of waking hours.
3 Capable of only limited self-care, confined to bed or chair more than 50% of waking hours.
4 Completely disabled. Cannot carry on any self-care. Totally confined to bed or chair.
5 Dead.
